# Recent advances in tumor-activated prodrug nanoparticles for cancer immunotherapy

**DOI:** 10.3389/fimmu.2026.1968703

**Published:** 2026-09-24

**Authors:** Guanran Wang, Kun Du, Dikai Sun, Shengyuan Tong, Yi Ren, Huicong Zhou

**Affiliations:** 1 China-Japan Union Hospital of Jilin University, Changchun, China; 2 School of Clinical Medicine, Tsinghua University, Beijing, China; 3 Key Laboratory and Engineering Laboratory of Lymphatic Surgery Jilin Province, China-Japan Union Hospital of Jilin University, Changchun, China

**Keywords:** drug delivery, immunotherapy, nanoparticles, prodrug, tumor

## Abstract

Numerous therapeutic agents have been demonstrated to initiate antitumor immune responses, thereby driving significant progress in cancer immunotherapy. However, clinical translation is severely limited by persistent challenges, including systemic toxicity, the immunosuppressive tumor microenvironment (TME), and low nanoparticle delivery efficiency. Tumor-activated prodrug nanoparticles (PDNPs) integrate prodrug chemistry with nanoscale delivery, enabling localized drug activation in response to tumor microenvironmental cues or exogenous stimuli. Therapeutic specificity is improved and off-target toxicity is mitigated by this strategy, providing a promising platform for safe and effective drug delivery. Consequently, these systems have attracted extensive research attention. In this review, the design strategies, release mechanisms, and immunotherapeutic applications of TME-responsive and exogenous stimulus-responsive PDNPs are systematically detailed, together with active-targeting and hybrid strategies that further improve tumor-selective delivery and activation. Beyond serving as delivery vehicles for immunomodulators, these PDNPs are utilized in synergy with chemotherapy, photothermal/photodynamic therapy, and sonodynamic therapy to trigger immunogenic cell death. Furthermore, dendritic cell maturation and T-cell infiltration are promoted, thereby remodeling the immune microenvironment, particularly when administered in combination with immune checkpoint inhibitors. Finally, the key challenges and considerations for the clinical translation of tumor-activated PDNPs are highlighted, with the aim of guiding further research progress.

## 1 Introduction

Cancer represents a complex systemic disease with a multifactorial etiology, characterized primarily by malignant cell proliferation, invasion, and metastasis. The rising global incidence and mortality rates impose a substantial burden on healthcare systems, thereby constituting a critical worldwide public health challenge (1). Fundamentally, tumorigenesis arises from the accumulation of genetic alterations, with studies indicating that 2 to 8 driver gene mutations are harbored by most human cancers (2). Conventional therapeutic modalities, including surgery, chemotherapy, radiotherapy, and their combinations, remain the standard of care. Tumors are physically removed via surgery, whereas cancer cells are eliminated by radiotherapy and chemotherapy through interference with their growth, proliferation, and survival. However, such interventions are frequently evaded by tumors through multiple mechanisms, such as acquired mutations and cancer stem cell-mediated drug resistance, ultimately resulting in recurrence, metastasis, and therapeutic failure (3). Furthermore, conventional therapies are frequently accompanied by severe adverse effects; anorexia, muscle atrophy, or central nervous system complications (such as seizures, headaches, cerebrovascular complications, vision loss, and myelopathy) may be caused by chemotherapy (4, 5). Immunotherapy has been established as a pivotal component of modern oncology due to the urgent need for durable, low-toxicity therapeutic options.

Remarkable clinical breakthroughs have been achieved by cancer immunotherapy, which employs strategies to mobilize the host immune system and activate endogenous immune cells to eliminate malignancies (6–8). The “cancer immunity cycle” typically comprises six critical steps: (1) tumor-antigen uptake and processing by antigen-presenting cells; (2) migration of antigen-presenting cells to lymphoid organs; (3) tumor-antigen presentation fine-tuned by co-stimulatory and co-inhibitory signals, leading to the activation of tumor-specific T cells and their differentiation into effector T cells within lymphoid organs; (4) egress of tumor-specific effector T cells from lymphoid organs into the peripheral blood and their trafficking to tumor sites; (5) tumor antigen recognition and tumor cell lysis; and (6) contraction of effector T cells along with the generation of tumor-specific memory T cells (9). A self-propagating cycle essential for eliciting effective antitumor immunity is established by these steps. However, immune escape is orchestrated by tumors primarily through the fostering of an immunosuppressive microenvironment, suppression of immune effector function, restriction of immune-cell infiltration, and the reduction of antigen recognition (10–13). To restore the cancer immunity cycle and reactivate antitumor responses, these escape mechanisms are generally targeted by current immunotherapeutic strategies (14). These strategies are broadly classified into five categories: (1) Tumor vaccines: tumor antigens are introduced into patients to enhance tumor immunogenicity, activate the host’s immune system, and induce immune responses, thereby controlling or eliminating tumors (15, 16); (2) Cytokine immunotherapy: cytokines are modulated to promote the maturation of immune cells (such as macrophages, natural killer cells, T lymphocytes, and dendritic cells), thereby enhancing their capacity to eliminate tumors (17–19); (3) Immune checkpoint blockade therapy: the immunosuppression of cytotoxic T lymphocytes by immune checkpoints is relieved, thereby restoring their ability to kill tumor cells (13, 20); (4) Cellular immunotherapy: patients are infused with immune cells that have been activated and engineered ex vivo, enabling them to exert potent antitumor effects *in vivo*, which has demonstrated promising clinical outcomes (21, 22); and (5) Small-molecule immunotherapy: small-molecule compounds are employed to modulate the activity of key enzymes or relevant receptors in immune pathways, an approach designed to improve the immune microenvironment, overcome immunosuppression, and activate antitumor immune responses (23, 24).

Despite the clinical approval and efficacy of various immunotherapies, their broader application remains hindered by significant barriers. Clinical data indicate that limited response rates to immunotherapy are observed in most solid tumors, typically ranging from 20% to 30% (25). Furthermore, immune-related adverse events are experienced by up to 37.7% of patients, which often necessitates the discontinuation of treatment due to severe side effects (26). To address these limitations, advanced nanoparticles have been engineered to selectively deliver therapeutic agents to tumor sites, thereby facilitating targeted cancer immunotherapy (27–29). Nanodrug delivery systems have been increasingly explored in cancer immunotherapy to improve tumor-directed delivery, promote immunogenic cell death, remodel the immunosuppressive TME, and interfere with tumor immune-evasion pathways (30). It was demonstrated in a meta-analysis by Wilhelm et al. that nanoparticle delivery efficiency remains remarkably low, with a median of only 0.7% of the administered dose ultimately reaching solid tumors. Manufacturing scalability and cost-effectiveness are significantly hampered by this inefficiency, while potential toxicity is exacerbated and therapeutic efficacy is limited (31). Although the development of numerous advanced nanoparticles has been prompted by this inefficiency, no significant improvement in the average delivery efficiency has been observed in recent years. Clinical translation is further impeded by the complex, heterogeneous physicochemical nature of the immunosuppressive tumor microenvironment (TME) and the physiological discrepancies between animal models and humans (32, 33).

Recently, tumor-activated prodrug nanoparticles (PDNPs) have emerged as a promising strategy for targeted cancer immunotherapy. Prodrugs represent pharmacologically inactive derivatives that undergo chemical transformation, wherein active parent drugs are released upon triggering by TME biomarkers (including enzymes, acidity, redox potential and hypoxia) or exogenous stimuli (such as ultrasound and light). The non-targeted toxicity associated with the therapeutic agents themselves is significantly mitigated by this mechanism (34). A representative strategy for constructing PDNPs involves the self-assembly of prodrug molecules through intermolecular covalent or noncovalent interactions, including hydrophobic effects, electrostatic interactions, and hydrogen bonding (35–37). Such self-assembled PDNPs can combine tumor-responsive drug activation with a reduced dependence on auxiliary carrier materials, thereby minimizing premature drug leakage and potentially decreasing carrier-associated burden (38). Furthermore, chemotherapeutics, photothermal agents, or sonosensitizers can be co-assembled with PDNPs, facilitating the integration of immunotherapy with multimodal treatments to overcome monotherapy resistance (39–42). In this review, strategies for achieving precise tumor targeting with PDNPs are systematically summarized, with a focus on their mechanisms in synergistic immunotherapy and potential for clinical translation, thereby providing a reference framework for the future design of PDNPs. The major activation mechanisms and design strategies of tumor-activated PDNPs are summarized in **Figure 1**.

**Figure 1:**
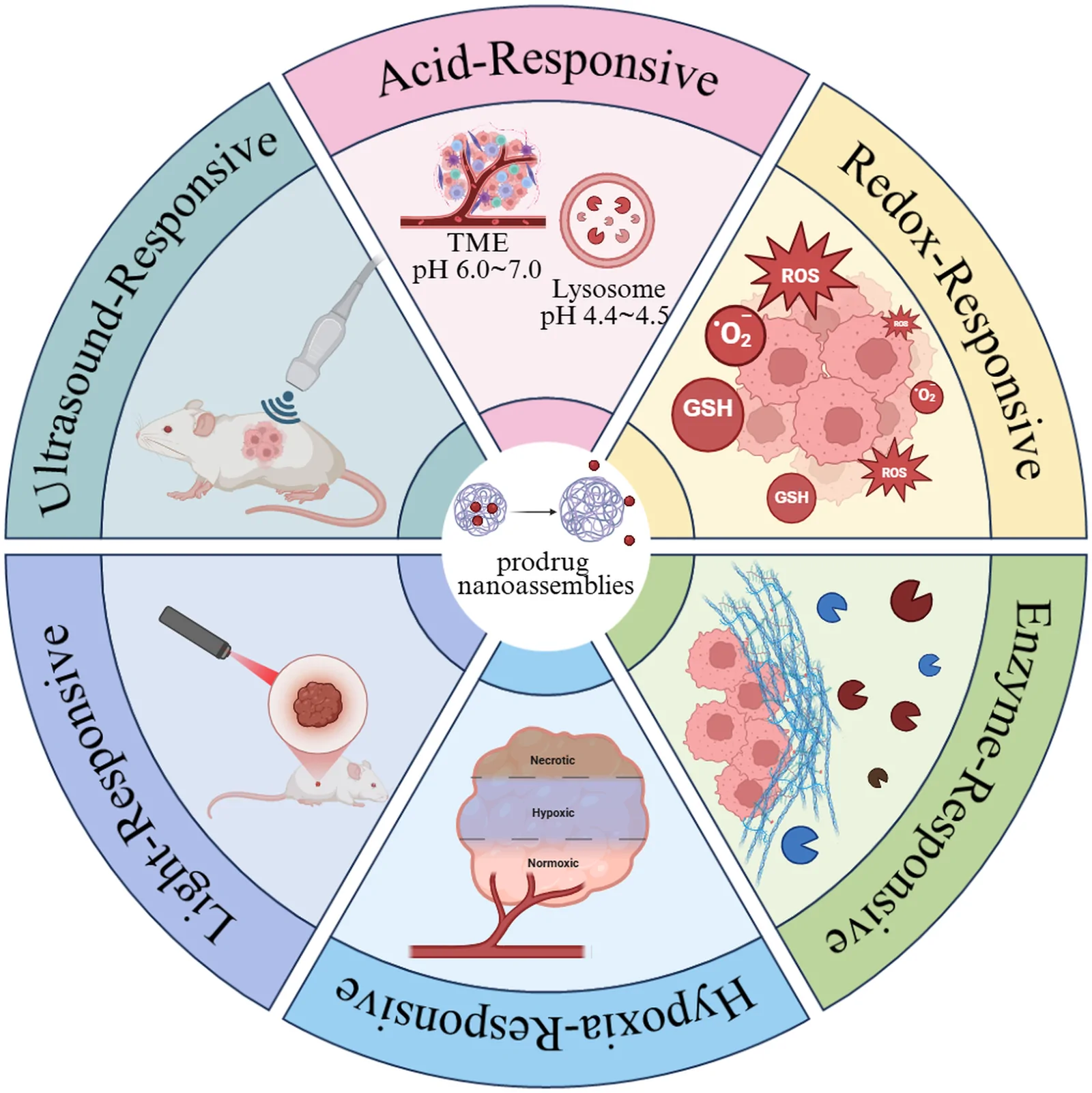
Stimuli-responsive mechanisms of PDNPs for cancer immunotherapy.

## 2 Tumor microenvironment-activated PDNPs

The tumor microenvironment is a complex ecosystem comprising a diverse array of immune cells, cancer-associated fibroblasts, endothelial cells, pericytes, and other tissue-specific cell types, such as adipocytes and neurons (43). Hypoxia is a hallmark of solid tumors, primarily resulting from aberrant tumor vasculature and elevated metabolic oxygen consumption during rapid cell proliferation (44, 45). Hypoxia-inducible factors, the best-characterized transcription factors in tumor hypoxia, regulate the transcription of numerous genes involved in every step of metastatic progression (46). Beyond hypoxia, aerobic glycolysis (the Warburg effect) constitutes another distinct metabolic feature, where the majority of glucose is converted to lactate even in the presence of oxygen. This metabolic reprogramming results in an acidic extracellular pH (6.5–6.9) (47, 48). As a critical intracellular antioxidant, glutathione (GSH) maintains intracellular redox homeostasis and participates in antioxidant defense. However, its elevated levels in tumor cells can significantly compromise therapeutic efficacy (49, 50). Certain overexpressed enzymes in the TME, such as matrix metalloproteinases and NAD(P)H:NQO1, play crucial roles in tumor invasion, migration, and proliferation (51). These unique physicochemical properties of the TME can be exploited by nanocarriers to achieve site-specific, controlled prodrug activation within tumor tissues (52). In this section, we categorize tumor-responsive PDNPs by their activation mechanisms and highlight how these designs elicit antitumor immune responses (**Table 1**).

**Table 1:** Summary of TME-responsive PDNPs for targeted cancer immunotherapy.

Type	Tumor-sensitive group	ICD inducer/immune activation strategy	Combination drug/modality	Cell lines/animal models	Therapeutic efficacy	Source
pH	Piperidine groups	GOD PTX	Anti-PD-1	Panc02; orthotopic Panc02-Luc/C57BL/6J	Median survival 80 d	(34)
	PEMA	DOX	NLG-919	4T1; bilateral 4T1/BALB/c	Distant tumor: complete inhibition	(54)
	Hydrazone bond	EPI	Anti-PD-1	4T1, MDA-MB-231; 4T1/BALB/c	TIR ~79%	(56)
	Benzoic imine bond Hydrazone bond	DOX	Anti-PD-1	CT26, 4T1; CT26/4T1-bearing mice	Tumor suppression ~74%/85%	(58)
	Schiff base bond	DOX	–	H22, M2 macrophages, HUVEC; H22-bearing mice	TIR 81%	(59)
	Hydrogen bonds	–	Anti-PD-L1	MC38; MC38/C57BL/6	Survival 40%	(60)
ROS	Thioether bond	DOX DHA	–	4T1; 4T1-bearing mice	TIR ~80%	(39)
	PPE	R848	Anti-PD-1	MB49, RAW264.7; MB49-bearing mice; patient organoids	TIR 98%	(61)
	Phenylboronic ester	CPT	DMXAA	B16-F10, 4T1; B16-F10/C57BL/6; orthotopic 4T1/BALB/c	Tumor burden ~1/6 of PBS	(62)
	Thioketal bond	SN38	Anti-PD-L1	CT26, HCoEpiC; subcutaneous/orthotopic CT26/BALB/c	CR 2/5	(63)
GSH	Disulfide bond	PTX	1MT	4T1, L929, DC2.4; 4T1/BALB/c	Apoptosis 74.3%	(65)
	Disulfide bond	Curcumin monomers T780	PTT PDT	AMC-HN-8, SCC7; AMC-HN-8/BALB/c nude	Tumor regression; recurrence reduced	(66)
Enzyme	RRK peptide	Verteporfin	PROTAC	GL261; subcutaneous/orthotopic GL261/C57BL/6	CR 100%	(37)
	Peptide	Semiconducting polymer	Ultrasound irradiation	4T1; bilateral 4T1/BALB/c	Primary/distant TIR 89%/90%	(68)
	FRRL peptide	DOX	Anti-PD-L1 antibody	CT26, H9C2; CT26-bearing mice	CR 100%	(35)
	DHPVGLLARD Peptide	R848	Cisplatin (Pt)	B16F10, RAW264.7; B16F10/C57BL/6	Survival 67%	(69)
	GALGLPG peptide	Pheophorbide a NLG-919	PDT	CT26; CT26/BALB/c (primary/abscopal)	Survival >80%	(70)
	Ester bond	Stapled KLA peptide	Anti-PD-L1	4T1; bilateral 4T1/BALB/c	Primary/distant TIR 88.0%/97.3%	(36)
	β-glucuronidase	IMDQ	–	RAW Blue reporter cells; IFNβ-reporter mice/draining LNs	IFN-β >3-fold increase	(72)
Hypoxia	Nitroaromatic linker	PTX	JQ1	4T1; 4T1-bearing mice	DC maturation +36.9%	(78)
	Nitroaromatic group	CPT	–	4T1, RAW264.7; orthotopic 4T1/BALB/c	Highest survival	(79)
	4-nitrobenzyloxy group	Cu(DTC)_2_ Curcumin	–	4T1; 4T1-bearing mice	4T1 viability 10.3%	(80)
	Ester bond	–	CA4‐NPs	CT26; CT26-bearing mice	TIR 98.1%	(81)
	Azo linker	DTC-Cu	–	4T1; 4T1/BALB/c	TIR 79.23%	(42)
	Azo linker	IMDQ	iRFA	H22, DC2.4; subcutaneous/bilateral/orthotopic H22/BALB/c	Tumor suppression 96.6%	(82)
	Azo linker	AQ4N	HIFU	4T1; bilateral 4T1/BALB/c	Survival 90%	(83)
	Azide group	IMDQ	CA4	4T1, MC38, CT26; corresponding mouse models	Tumor eradication without recurrence	(84)
	Azide group	R848	Anti-PD-1	4T1, CT26, MC38; BALB/c/C57BL/6 mice	TSR 99.4% (4T1); 4/5 cured (CT26)	(85)

TME, tumor microenvironment; PDNPs, prodrug nanoparticles; ICD, immunogenic cell death; ROS, reactive oxygen species; GSH, glutathione; GOD, glucose oxidase; PTX, paclitaxel; PD-1, programmed cell death protein 1; BA, betulinic acid; Ce6, chlorin e6; PD-L1, programmed death-ligand 1; PDT, photodynamic therapy; PEMA, 2-(pentamethylene imino)ethyl methacrylate; DOX, doxorubicin; NLG-919, 1-cyclohexyl-2-(5H-imidazo[5,1-a]isoindol-5-yl)ethanol; OXP, oxaliplatin; CD47, cluster of differentiation 47; EPI, epirubicin; DHA, dihydroartemisinin; PPE, phenylboronic acid pinacol ester; R848, resiquimod; CPT, camptothecin; DMXAA, 5,6-dimethylxanthenone-4-acetic acid; SN38, 7-ethyl-10-hydroxycamptothecin; 1MT, 1-methyl-tryptophan; T780, TPGS-modified IR780 photothermal agent; PTT, photothermal therapy; PROTAC, proteolysis-targeting chimera; Pt, platinum; NPs, nanoparticles; MSA-2, non-nucleotide stimulator of interferon genes agonist; KLA, pro-apoptotic KLA peptide; IMDQ, imidazoquinoline; JQ1, bromodomain and extraterminal domain inhibitor JQ1; DTC, diethyldithiocarbamate; CA4, combretastatin A4; iRFA, incomplete radiofrequency ablation; AQ4N, banoxantrone; HIFU, high-intensity focused ultrasound; Azo, azobenzene. Abbreviations: TIR, tumor inhibition rate; CR, complete regression.

### 2.1 pH-responsive PDNPs

pH-responsive PDNPs are typically engineered by incorporating pH-sensitive moieties, such as amines or carboxyl groups, into the prodrug architecture (34). Protonation of these moieties within the acidic TME triggers nanostructure disassembly, thereby enabling tumor-specific drug release. A pH-responsive micellar nanoreactor, pro-PTX NRs, was developed by incorporating piperidine units (pKa ≈ 6.75). This design enables the nanoreactor to respond to the weakly acidic TME (pH ≈ 6.5), where protonation of the piperidine groups induces nanostructural swelling. This swelling restores the encapsulated glucose oxidase (**Figure 2**), which catalyzes glucose oxidation to generate hydrogen peroxide (H_2_O_2_), subsequently cleaving the oxalate ester linkages to release active paclitaxel. Approximately 60% of the prodrug was activated within 48 hours *in vitro*. In an orthotopic pancreatic cancer model, combining pro-PTX NRs with a PD-1 inhibitor significantly suppressed tumor growth and extended the median survival of mice from 31 days (control group) to 80 days. The nano-system COS-BA/Ce6 NPs, co-assembled from a chitosan oligosaccharide-modified betulinic acid prodrug and the photosensitizer Chlorin e6 (Ce6), employs synergistic chemo-photodynamic therapy to induce immunogenic cell death (ICD) (53). This system responds to the weakly acidic TME through protonation of the amino groups on chitosan oligosaccharide, which disrupts the hydrogen-bonding network within the nanoparticles and triggers responsive disassembly, thereby releasing the chemotherapeutic component betulinic acid and the photosensitizer Ce6. In a 4T1 breast cancer model, combined with anti-PD-L1 immunotherapy, this system achieved an inhibition rate of 80.3% against the primary tumor and successfully activated a systemic anti-tumor immune response, suppressing the growth of distant non-irradiated tumors.

**Figure 2:**
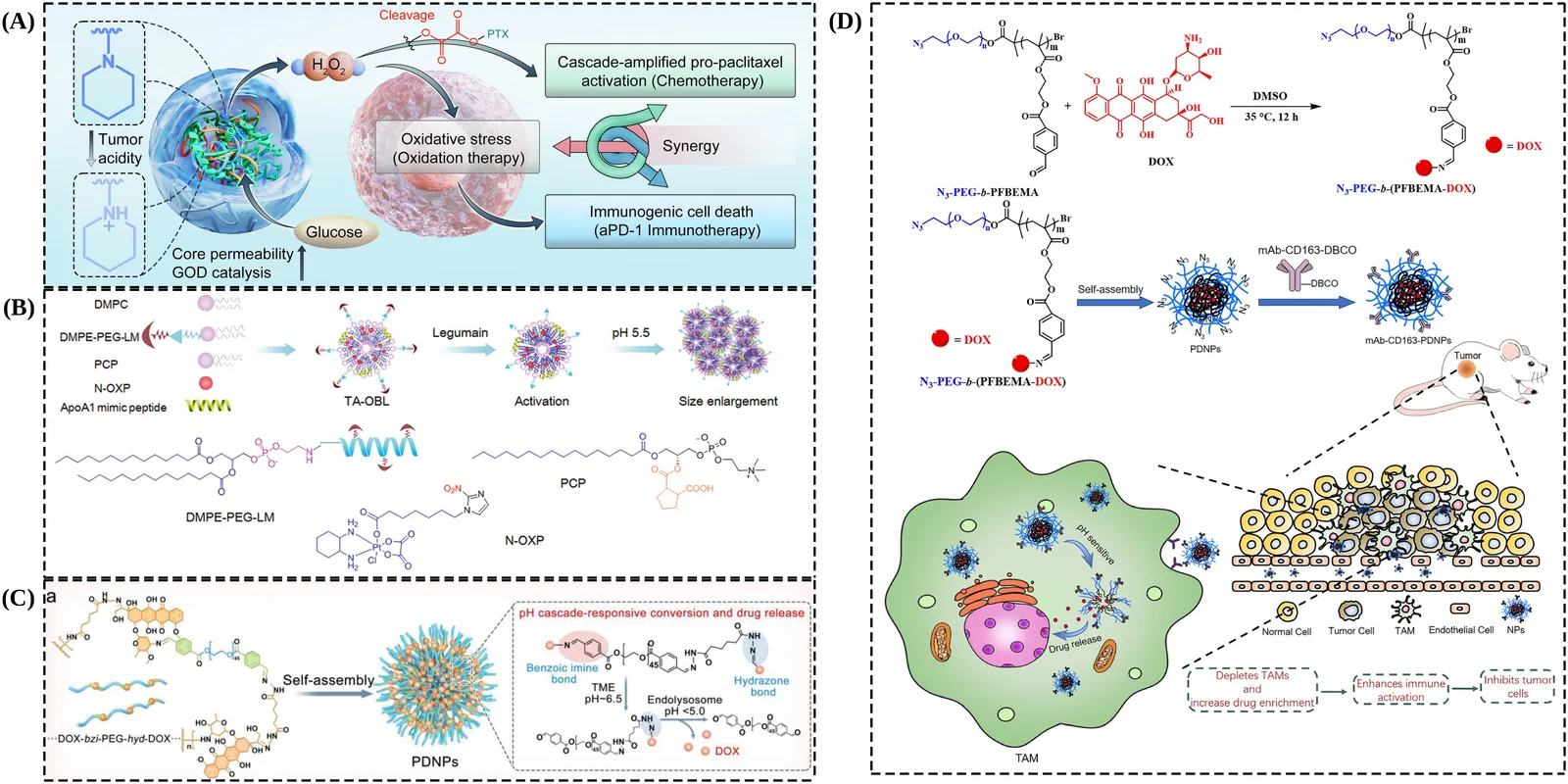
**(A)** Activated GOD catalyzes glucose oxidation to generate ROS, triggering prodrug cleavage and release of active paclitaxel. The cascade induces cytotoxicity and immunogenic cell death, enabling synergistic oxidation–chemo–immunotherapy (34). This image is available under the CC BY 4.0 license. **(B)** Schematic illustration of cancer-accessing TA-OBL to boost antitumor immune responses and synergize ICBs-mediated immunotherapy. TA-OBL would be activated by highly expressed legumain in tumor to promote their intratumoral permeation and accessibility to cancer cell fractions. Afterward, TA-OBL undergoes size-enlargement in intracellular acidic environments to prolong their tumor retention (55). Copyright 2020, Wiley. **(C)** Chemical structure and fabrication of PDNPs and the mechanism of pH cascade-responsive conversion and drug release (58). Copyright 2022, Wiley. **(D)** Schematic illustration of synthesis and self-assembly of mAb-CD163^+^-PDNPs nanoparticles modified by CD163^+^ monoclonal antibody, which can target and deplete tumor-associated macrophages (59). This image is available under the CC BY 4.0 license.

Beyond merely leveraging protonation in the acidic milieu to trigger the disassembly of PDNPs, more advanced nanoscale prodrug systems also enable precise regulation of PDNPs size, thereby prolonging their retention time within tumor tissues. Japir et al. constructed a β‐gal@PSLC system, in which the protonation of PEMA units (pKa ≈ 6.91) triggers the swelling of the polymersomes in the acidic TME, increasing vesicle size from ~100 nm to 200 nm (54). Consequently, fluorescence retention in tumors remained at 36.2% after 6 days, significantly surpassing the 3.5%–13.1% observed in non-swelling controls. Through multiple administrations, β‐gal@PSLC achieved long‐term local prodrug activation within the tumor. This not only markedly suppressed the growth of primary tumors but also completely inhibited untreated distant metastatic foci. Systemically, it enhanced the infiltration of CD8^+^ T cells, reduced the proportion of regulatory T cells, and lowered the Kyn/Trp ratio in the tumor tissue, thereby realizing synergistic chemo‐immunotherapy with minimal systemic toxicity. The tumor-activated, size-enlargeable bioinspired lipoprotein system, TA-OBL, employs a pH-sensitive phospholipid to achieve intracellular size expansion and controlled drug release (55). Under the acidic lysosomal condition of pH 5.5, the particle size of TA-OBL rapidly increased from 22.05 nm to over 300 nm, promoting intracellular retention and aggregation of the nanoparticles (**Figure 2**). Simultaneously, the release rate of the hydrophobic oxaliplatin prodrug reached approximately 80% within 24 hours, significantly higher than the about 35% observed under neutral conditions. This pH-responsive release mechanism effectively potentiates oxaliplatin-induced ICD. Furthermore, the system significantly promoted calreticulin (CRT) exposure, HMGB1 release, and ATP secretion upon acidic triggering, thereby enhancing dendritic cell maturation and T-cell infiltration, providing a mechanistic basis for synergy with immune checkpoint inhibitors.

Novel delivery strategies facilitate controlled drug release through the cleavage of acid-labile bonds. The POEGDE@A system uses acid-labile hydrazone bonds to covalently conjugate epirubicin (EPI) to its dendritic termini (56). Under weakly acidic tumor microenvironmental conditions (pH 5.4), the cumulative release of EPI exceeded 40% within 12 hours and approached 80% by 48 hours. In contrast, the release rate was less than 30% under physiological pH 7.4. Simultaneously, the adenosine receptor antagonist AB928 was co-loaded via hydrophobic interactions, enabling the synchronized release of both EPI and AB928 within the TME. This design facilitates the induction of ICD while concurrently reversing adenosine-mediated immunosuppression. In the 4T1 tumor-bearing mouse model, monotherapy with POEGDE@A achieved approximately 72% tumor growth inhibition, which was significantly superior to the 49.6% tumor growth inhibition observed with free EPI monotherapy. Furthermore, the tumor growth inhibition was further elevated to 79% when POEGDE@A was combined with an anti-PD-1 antibody. Similarly, the prodrug-based nanoparticles achieved targeted drug release through hydrazone bond hydrolysis in the acidic TME, with the doxorubicin (DOX) release rate at pH 5.0 being approximately 3.2 times higher than that under physiological pH 7.4 (57). Upon combined laser irradiation, prodrug-based nanoparticles exhibited a 2.8-fold increase in CRT exposure on the cell membrane and a 3.1-fold enhancement in ATP release, indicating significantly improved ICD induction. In 4T1 tumor-bearing mice, prodrug-based nanoparticles combined with PD-1 antibody led to an 86.7% inhibition rate of distant tumor volume, a 41.5% decrease in the proportion of Ly6G^3^ myeloid‐derived suppressor cells (MDSCs) in the spleen, and a 2.4-fold increase in intratumoral CD8^+^ T cell infiltration. These studies exemplify the parallel development and synergistic potential of complementary strategies: targeting immunosuppression and boosting immune activation.

To further heighten sensitivity to the acidic TME, a cascade-activated supramolecular PDNP was engineered using dual acid-responsive linkages, ultra-pH-sensitive benzoic imine and hydrazone bonds (58). Through a stepwise size shrinkage and dePEGylation strategy, PDNPs enable precise drug delivery and release within the TME, thereby efficiently inducing gasdermin E-mediated pyroptosis and enhancing antitumor immunity. At physiological pH, PDNPs maintain a size of ~130 nm, with the polyethylene glycol (PEG) shell providing “stealth” properties to prolong circulation and promote tumor accumulation. In the acidic extracellular environment (pH ≈ 6.5), protonation of the benzoic imine bond triggers PEG detachment and size reduction to about 38 nm, significantly improving deep tumor penetration. Upon entering more acidic endolysosomes (pH ≈ 5.0), cleavage of the hydrazone bond leads to complete dissociation of PDNPs into ultrasmall units of about 8 nm (**Figure 2**), ensuring precise DOX delivery to the nucleus. This cascade pH-responsive mechanism results in cumulative DOX release rates of 43.4% and 86.6% at pH 6.5 and 5.0, respectively, markedly higher than the 18.6% observed at physiological pH. In CT26 and 4T1 tumor models, PDNPs combined with anti-PD-1 therapy achieved tumor inhibition rates of approximately 74% and 85%, respectively, demonstrating significant synergistic immunotherapeutic efficacy. Yang et al. designed mAb-CD163^+^-PDNPs in which DOX was conjugated to the polymeric carrier via a pH-sensitive Schiff base bond (59). Under lysosomal pH 5.0 conditions, the bond underwent rapid hydrolysis, releasing approximately 80% of DOX within 76 hours. The nanoparticles specifically depleted CD163^+^ M2-type tumor-associated macrophages, thereby remodeling the immunosuppressive TME (**Figure 2**). In an H22 hepatoma model, this strategy achieved a tumor inhibition rate of 81%.

Unlike conventional systems relying on covalent bond cleavage, the NOTAb nanocomplex achieves precise anti-PD-L1 release via a pH-dependent hydrogen bond dissociation mechanism (60). In the acidic TME at pH 6.5, the hydrogen bonding between tannic acid and anti-PD-L1 is significantly weakened, leading to efficient antibody release from the complex, with a release rate more than 9-fold higher than that at physiological pH 7.5. Flow cytometry analysis further confirmed that the anti-PD-L1 released under acidic conditions restored its binding capability to MC38 tumor cells, with efficiency comparable to that of free antibody, whereas it was markedly reduced under neutral conditions. These findings validate the feasibility of exploiting the acidic TME for controlled PDNPs activation and release.

Collectively, pH-responsive PDNPs offer a relatively versatile strategy because acidic conditions can regulate drug release through multiple mechanisms, including protonation-induced nanostructural transformation, cleavage of acid-labile covalent bonds, and disruption of noncovalent interactions. The studies discussed above further indicate that pH responsiveness can be coupled with size transformation, prolonged tumor retention, or stepwise intracellular release to improve spatial control of drug activation. Nevertheless, the term “pH-responsive” encompasses mechanistically distinct activation environments that should not be considered equivalent. Systems triggered at approximately pH 6.5 primarily exploit extracellular tumor acidity, whereas those requiring pH 5.0–5.5 depend more strongly on acidic endosomal or lysosomal compartments after cellular uptake. Therefore, responsiveness under strongly acidic conditions does not necessarily indicate selective activation within the extracellular tumor microenvironment. In addition, heterogeneity in tumor acidity may lead to variable activation efficiency among different tumor regions and models. Multistage or cascade-responsive designs can partially improve this spatial selectivity by sequentially exploiting extracellular and intracellular acidity, but the additional structural components may also increase formulation complexity. Thus, the optimal pH-responsive PDNP should balance sufficient activation sensitivity with structural simplicity and clearly distinguish whether its principal activation site is the extracellular tumor microenvironment or the intracellular acidic compartment.

### 2.2 Redox-responsive PDNPs

Redox-responsive PDNPs represent a key technological strategy to address the challenges of low delivery efficiency and high systemic toxicity in immunotherapy. These PDNPs achieve tumor-specific drug activation and release by responding to the abnormally high concentrations of reactive oxygen species (ROS) or the reducing agent GSH within the TME. Designs that target the high-ROS TME primarily aim to achieve spatiotemporally controlled immune activation and synergistic therapeutic effects. Han et al. constructed a ROS-sensitive dihydroartemisinin (DHA) prodrug nanoparticle (39). The nanoparticle utilizes the high-ROS microenvironment within tumor cells to trigger drug release, in which the thioether bond-linked DHA prodrug cleaves under ROS conditions, enabling spatiotemporally synchronized release of DOX and DHA. On one hand, the released DOX targets the endoplasmic reticulum, exacerbating endoplasmic reticulum stress and significantly enhancing ICD, as evidenced by an approximately 2-fold increase in CRT exposure and markedly elevated release of HMGB1 and ATP. On the other hand, the synchronously released DHA downregulates PD-L1 expression by inhibiting STAT3 phosphorylation and the NF-κB pathway, reducing PD-L1 levels in tumor cells by approximately 50%. In a 4T1 tumor-bearing mouse model, this system achieved nearly 80% tumor inhibition rate, significantly increased intratumoral infiltration of CD8^+^ T cells, and concurrently reduced the proportion of regulatory T cells. Similarly, Geng et al. developed ROS-responsive R848 PDNPs (61). The PDNPs were synthesized by covalently conjugating the Toll-like receptor 7/8 (TLR7/8) agonist R848 to phenylboronic acid pinacol ester via a succinic anhydride linker, forming the prodrug R–P. This prodrug structure enables specific oxidative cleavage of the phenylboronic acid pinacol ester moiety by H_2_O_2_ in the TME with elevated ROS, resulting in the release of active R848 (**Figure 3**). The released R848 activates the TLR7/8 signaling pathway, thereby reprogramming pro-tumoral M2-type tumor-associated macrophages into anti-tumoral M1-type macrophages and subsequently reversing the immunosuppressive TME. This PDNP exhibited significant ROS-responsive drug release characteristics, with approximately 95% of R848 released within 24 hours under 200 μM H_2_O_2_, compared to only about 10% release in the absence of H_2_O_2_. In an MB49 bladder cancer model, tumor accumulation of R-P@APM-DPs was 1.8-fold higher than that of non-targeted nanoparticles. Monotherapy with R-P@APM-DPs achieved a 90% tumor inhibition rate. Combination therapy with an anti-PD-1 antibody further increased the inhibition rate to 98% and significantly prolonged survival in mice.

**Figure 3:**
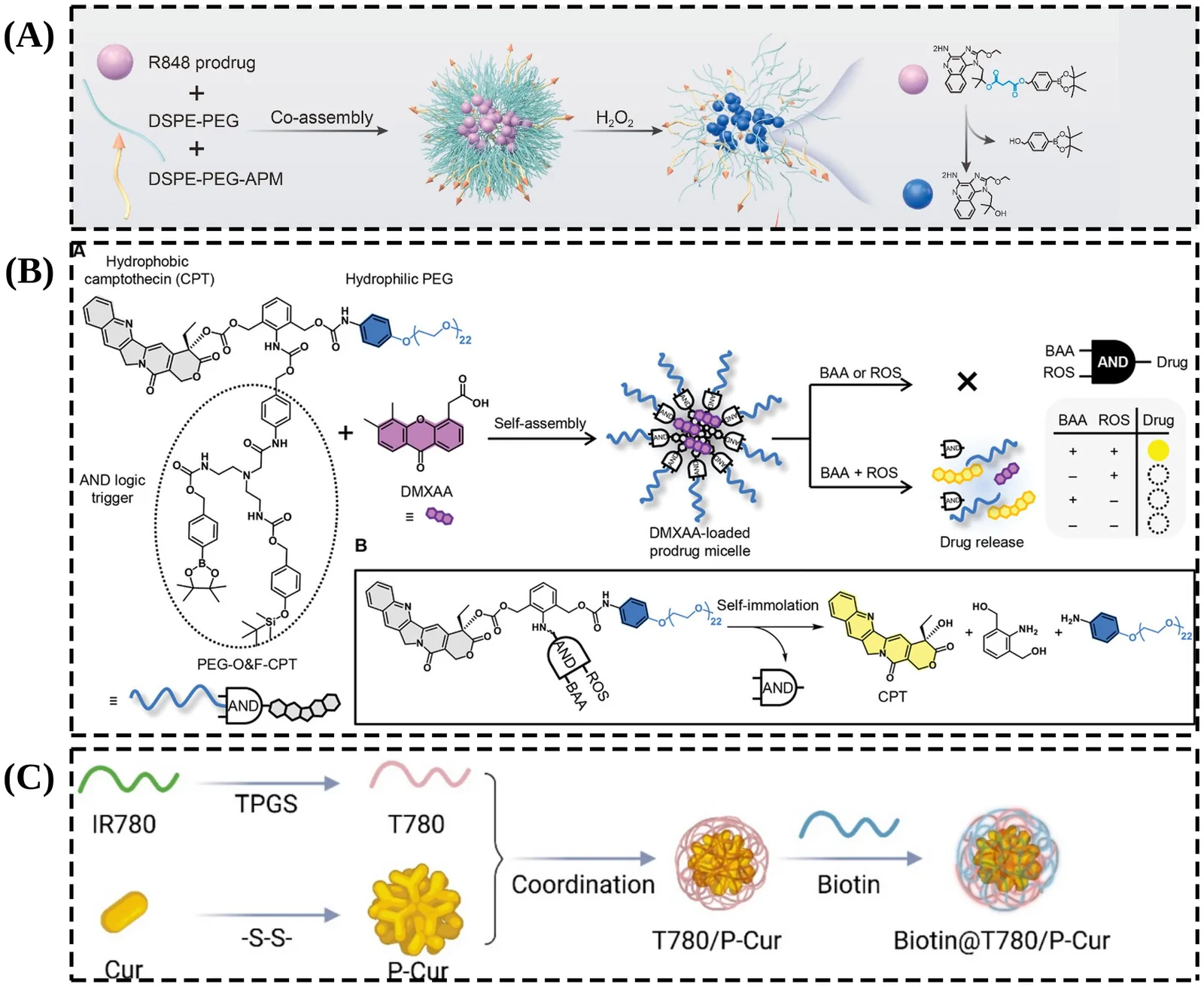
**(A)** Preparation and ROS-responsive release of ROS-responsive R848 PDNPs (61). Copyright 2026, Elsevier. **(B)** Schematic illustration of the formation of the DMXAA-loaded AND logic-gated prodrug micelle (DMXAA@PEG-O&F-CPT) through self-assembly and its AND logic drug release. This AND logic-controlled release system uses tumor-enriched boramino acids and ROS as inputs, micelle dissociation as the Boolean logic-based algorithm, and drug release as a single output. It functions as an AND gate because both triggers are required to completely degrade the micelle via sequential self-immolations (62). Copyright 2023, Wiley. **(C)** Preparation of Biotin@P-Cur/T780 NPs (66). This image is available under the CC BY 4.0 license.

To further enhance tumor selectivity, advanced logic-gated systems activated by multiple stimuli have been employed in the construction of nanoscale prodrugs. An “AND” logic-gated prodrug micelle was designed to release drugs only upon simultaneous response to tumor-enriched boramino acids and elevated levels of ROS (62). The micelle employs a phenylboronic ester as the ROS-responsive unit, which undergoes desilylation upon ROS exposure, thereby triggering a cascade of self-immolation reactions and ultimately achieving controlled drug release (**Figure 3**). In the presence of both boramino acids and H_2_O_2_, the micelle achieved nearly 100% release of camptothecin (CPT), with a release efficiency approximately 200-fold higher than that under single-stimulus conditions. Furthermore, after efficient encapsulation of the stimulator of interferon genes (STING) agonist DMXAA via π–π stacking and hydrophobic interactions, the DMXAA@PEG-O&F-CPT micelles significantly activated immune responses within tumors, elevating IFN-β secretion to 59–70 times that of other groups. In another study, a cascaded self-uncloaking dual-prodrug nanoassembly was constructed with an outer shell composed of DSPE-TK-PEG-CH1055 containing a thioketal linker, enabling precise spatiotemporal control of drug release (63). Under the high-ROS conditions of tumor tissue, the shell undergoes cleavage, leading to 75% release of CH1055 within 24 h and initiating near-infrared-II imaging, thereby achieving “diagnosis-first” visualization. Subsequently, the core further releases the chemotherapeutic agent SN38 and the TIM3-targeting CRISPR/Cas13a gene-editing system in deeper hypoxic regions, which synergistically activates the cyclic GMP-AMP synthase (cGAS)–STING pathway and reprograms the immunosuppressive microenvironment. In a PD-L1-resistant colorectal cancer mouse model, this ROS-responsive cascaded release system, combined with anti-PD-L1 therapy, achieved a complete remission rate of 40%. To address the challenge of low drug loading capacity in clinical translation, recent research has conjugated paclitaxel (PTX) with a phenylboronate ester (PBE) linker, which can be oxidatively cleaved in the TME characterized by high levels of ROS, to synthesize a PTX-PBE prodrug (64). This design enables the specific release of active PTX within tumor cells. The PTX-PBE prodrug co-assembles with a combretastatin A4 (CA4)-conjugated polymeric conjugate possessing vascular-disrupting activity, mPEG-PCA4 (abbreviated as PCA4), to form nanoparticles. This system achieves an exceptionally high drug loading capacity of up to 99%. In the high-ROS TME, the PTX-PBE prodrug is activated to release PTX, which works synergistically with CA4 released from the carrier itself. This combination simultaneously kills tumor cells and disrupts tumor vasculature. In a colorectal cancer model, this nanomedicine demonstrated a remarkable tumor suppression rate of 87.2%, while effectively circumventing the cardiotoxicity associated with free CA4.

Corresponding to ROS responsiveness, the super-high concentration of reductive GSH within tumor cells represents another critical stimulus-responsive target. Prodrugs featuring GSH-sensitive linkers (e.g., disulfide bonds) are rapidly cleaved and release drugs via reduction within the tumor intracellular environment. This process concurrently depletes intracellular GSH and disrupts redox homeostasis, potentially triggering unique immunogenic effects. For instance, self-carrier nanoparticles (PSMT NPs) were prepared by co-assembling a redox-responsive paclitaxel prodrug (PSSP), which contains a disulfide bond, with the indoleamine 2,3-dioxygenase (IDO) inhibitor 1-methyl-tryptophan (65). These nanoparticles enable precisely triggered drug release in response to the reductive TME, characterized by high GSH concentrations. The disulfide bond in PSSP can be efficiently cleaved, leading to the synchronous release of active PTX and 1-methyl-tryptophan. A more advanced strategy involves the simultaneous regulation of GSH concentration in the TME via GSH-responsive mechanisms. Shen et al. constructed a prodrug nanoplatform (Biotin@P-Cur/T780 NPs), in which curcumin monomers were polymerized via disulfide bonds to form the polymeric prodrug P-Cur (66) (**Figure 3**). These disulfide bonds undergo specific cleavage in the high-GSH environment of tumor cells, releasing active curcumin and concurrently depleting intracellular GSH. In a subcutaneous xenograft model of head and neck cancer using AMC-HN-8 tumor-bearing BALB/c nude mice, this prodrug nanoplatform significantly inhibited tumor growth, demonstrating superior efficacy compared to free drug groups and non-targeted nanoparticle groups. *In vitro* tumor stem cell sphere formation assays revealed that the platform suppressed the sphere-forming ability of SCC7 cells and markedly downregulated the expression of stemness markers such as ZEB1 and Nanog. To increase the drug loading capacity of nano-co-delivery systems and enhance synergistic efficacy and therapeutic outcomes, Zhou et al. developed a prodrug composed of PTX and CA4 (67). PTX was conjugated to CA4 via either an alkyl chain or a reduction-responsive disulfide linkage through simple esterification reactions, enabling the synchronous and efficient delivery of both agents. The resulting prodrug-designed nanoparticles achieved near-quantitative drug loading, with an exceptionally high drug loading capacity of 99% and a drug loading efficiency approaching 100%. In a simulated TME (pH 6.8 containing 20 mM GSH), the cumulative drug release reached 68.8% within 72 hours, which was significantly higher than the release under physiological conditions (pH 7.4). In a CT26 colon carcinoma mouse model, the PDNPs treatment group exhibited a markedly high tumor suppression rate of 92.8% without causing significant body weight loss. Both the efficacy and safety profiles were substantially superior to those of the free drug combination group and the single prodrug group.

Overall, redox-responsive PDNPs provide two complementary design opportunities by exploiting either oxidative or reductive conditions. ROS-responsive systems can couple drug activation with oxidative stress and can be further integrated with cascade or logic-gated designs to enhance activation selectivity, whereas GSH-responsive systems are particularly suited for intracellular drug release through reduction-sensitive linkages such as disulfide bonds. In addition to triggering payload release, some redox-responsive designs can simultaneously perturb tumor redox homeostasis, thereby contributing to therapeutic amplification. However, the efficacy of these systems is closely dependent on the local redox state, and variations in ROS or GSH levels may lead to heterogeneous activation among tumors or within different tumor regions. Moreover, GSH-responsive activation generally occurs after cellular internalization and therefore primarily improves intracellular release rather than tumor-tissue localization itself. Multi-trigger or logic-gated systems may enhance activation specificity, but this benefit is accompanied by increased molecular complexity and more demanding formulation control. Accordingly, the translational value of redox-responsive PDNPs depends not only on achieving stimulus-sensitive drug release, but also on ensuring that the selected redox trigger is sufficiently abundant, spatially appropriate, and compatible with a reproducible nanoparticle design.

### 2.3 Enzyme-responsive PDNPs

Cathepsin B (CatB), a lysosomal cysteine protease frequently overexpressed in various tumor cells, serves as a classical target for designing tumor-specific prodrugs. Capitalizing on this target, Jang et al. reported a Cathepsin B-activatable PROTAC nanoassembly (PN_PTP1B_) for photodynamic PTP1B proteolysis (37). In this design, a PTP1B-targeting PROTAC molecule is covalently linked to the photosensitizer Verteporfin (VPF) via a CatB-specific cleavable RRK peptide sequence. Driven by intermolecular π–π stacking interactions among the aromatic segments of VPF, the conjugate spontaneously self-assembles into uniform nanoparticles with an average diameter of 87.79 ± 1.25 nm. This supramolecular assembly strategy eliminates the need for additional carrier materials and achieves a remarkably high drug loading capacity of 88.6%. Similarly, Wu et al. designed a CatB-responsive exosome-inhibiting polymeric sonosensitizer (68). This system comprises a semiconducting polymer backbone (serving as the sonosensitizer) and PEG side chains conjugated with a prodrug-type exosome inhibitor, DPTIP, via a CatB-cleavable linkage. In the TME with high CatB expression, the prodrug is activated, releasing free DPTIP which inhibits neutral sphingomyelinase 2, thereby blocking tumor exosome biogenesis. Compared to the control without the inhibitor (CPS + US), the exosome-inhibiting polymeric sonosensitizer + US treatment reduced serum exosome levels by 49% and completely prevented lung metastasis. Another strategy developed a CatB-responsive DOX prodrug nanoparticle (CatB-NPs) (35). This prodrug was formed by covalently conjugating the self-assembling peptide Phe-Arg-Arg-Leu to DOX, which is specifically cleaved within CatB-overexpressing tumor cells to release active DOX (**Figure 4**). Compared to free DOX, CatB-NPs showed an approximately 9.4-fold higher IC_50_ in CT26 cancer cells, while their cytotoxicity against normal and immune cells was reduced by about 500–800 fold. In the CT26 tumor model, the *in vivo* tumor-targeting fluorescence intensity of CatB-NPs was about 1.33-fold higher than that of free DOX, and the ex vivo tumor fluorescence was about 1.49-fold stronger. When combined with an anti-PD-L1 antibody, CatB-NPs achieved 100% complete tumor regression and established long-term immunological memory capable of resisting tumor rechallenge. Although these studies employed different strategies, they all demonstrated significant efficacy, highlighting the important potential of cathepsin B-responsive nanoprodrugs in tumor immunotherapy.

**Figure 4:**
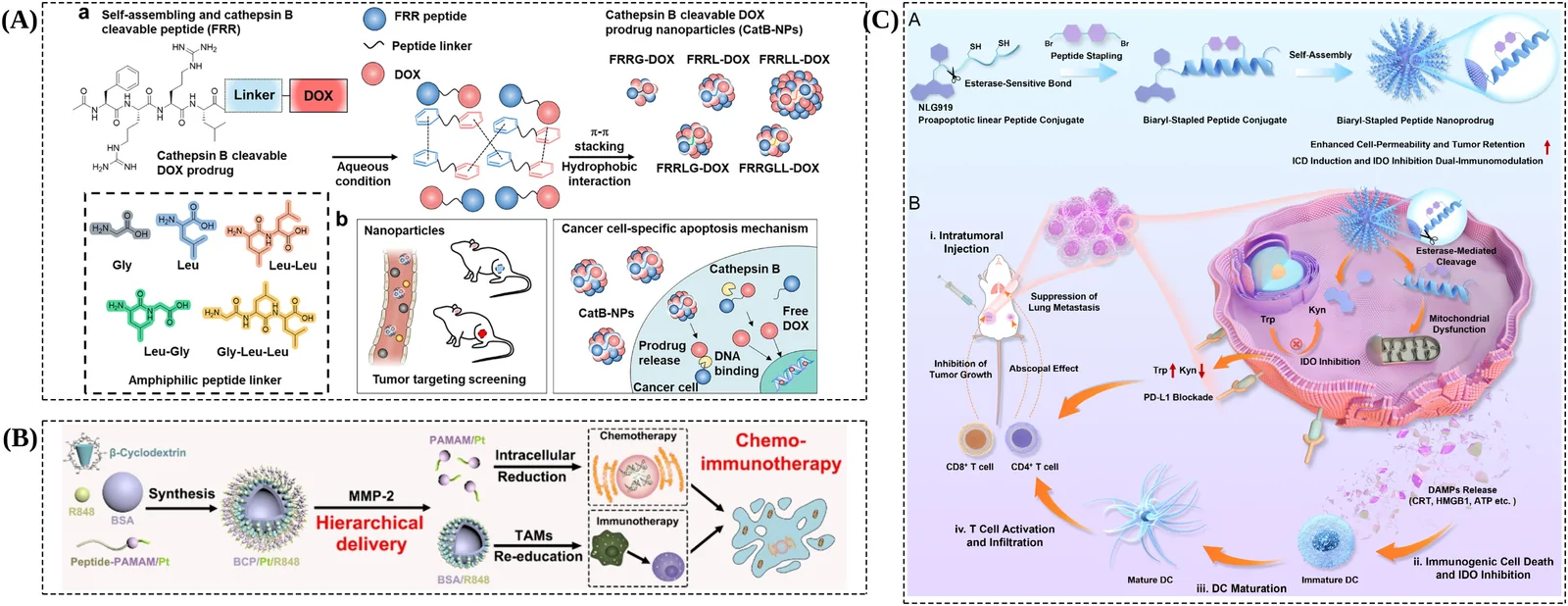
**(A)** Chemical structures of five self-assembling and cathepsin B-cleavable peptides (FRRX) conjugated DOX prodrugs. Each peptide sequence contains self-assembling peptide (F), cathepsin B-cleavable peptide (RR), and lysosomal enzyme-cleavable peptide linker (X = G, L, LL, GL, or LG). The different amphiphilicity of prodrug can significantly influence the nanoparticle structure via intermolecular hydrophobic interaction and π–π stacking. The optimal nanoparticle structure of CatB-NPs can be assayed *in vitro* and *in vivo* as the lead formulation based on their superior nanoparticle stability and tumor-specific accumulation profile in tumor-bearing mice. The optimal CatB-NPs can accumulate efficiently at targeted tumor tissue via the nanoparticle-derived EPR effect and then induce the cancer cell-specific ICD via cathepsin B-specific cleavage mechanism in cathepsin B-overexpressed cancer cells (35). Copyright 2025, Wiley **(B)** Synthesis routes and hierarchical delivery schematic illustration of MMP-2/GSH-responsive BCP/Pt/R848 nanobomb for tumor chemo-immunotherapy and re-education of tumor-associated macrophages (69). This image is available under the CC BY 4.0 license. **(C)** Molecular engineering of the dual-immunomodulatory stapled peptide nanoprodrug. Illustration of the proposed mechanism of nanoprodrug-mediated synergistic immunotherapy by concurrent ICD induction, IDO inhibition, and PD-L1 blockade (36). Copyright 2025, American Chemical Society.

Matrix metalloproteinases are key extracellular enzymes involved in tumor invasion and metastasis, and their specific substrate peptides are frequently utilized as responsive linkers in prodrug design. For instance, Zheng et al. engineered a cascade delivery nanosystem based on MMP-2 enzymatic responsiveness (69). This system employed bovine serum albumin (BSA) as the core. An MMP-2 cleavable peptide (Asp-His-Pro-Val-Gly-Leu-Leu-Ala-Arg-Asp) was used to covalently conjugate PAMAM dendrimers loaded with a cisplatin prodrug (Pt(IV)). Simultaneously, β-cyclodextrin-modified BSA was utilized to encapsulate the immunomodulator R848 (**Figure 4**). In the TME characterized by high MMP-2 expression, the peptide linker is specifically cleaved, releasing the PAMAM/Pt component with a size of approximately 120 nm. The cumulative release rate of PAMAM reached 55.3% within 4 hours. Pharmacokinetic analysis revealed that, compared to free cisplatin, the PDNPs (BCP/Pt/R848) exhibited a significantly prolonged blood circulation time. In a B16F10 tumor-bearing mouse model, treatment with this system reduced the tumor volume to the pre-administration level compared to the control group and decreased lung metastatic nodules by approximately 70%. A similar MMP-responsive strategy has also been employed to construct multifunctional synergistic systems. For instance, Hou et al. designed Boolean logic-based enzyme-responsive PDNPs by integrating an extracellular matrix metalloproteinase-2/9 (MMP-2/9)-responsive “E-YES” logic gate that is activated by the overexpressed MMP-2/9 in the TME (70). This enables enzyme-controlled nanoparticle dissociation and prodrug activation. Specifically, MMP-2/9 cleaves the enzyme-sensitive peptide sequence GALGLPG linked to the PEG corona, triggering PEG detachment and thereby enhancing nanoparticle accumulation and cellular uptake in tumor tissues. After pretreatment with MMP-2/9, the cellular uptake of PGDNP nanoparticles was increased by 6.1-fold compared to the non-responsive control PDNPs, and by 3.0-fold compared to untreated PGDNP. In a CT26 colorectal tumor-bearing mouse model, PGDNP exhibited 2.8-fold higher fluorescence intensity at the tumor site than PDNPs, along with a 2.4-fold increase in NLG-919 accumulation in the tumor.

Esterase is also overexpressed in the TME, making the use of ester bonds as responsive linkers a simple yet effective strategy. Syeda et al. covalently conjugated MSA‐2 with NLG‐919 via an ester bond to form MSA‐NLG, which was then encapsulated with DSPE‐mPEG2K into nanoparticles for co‐delivery of the STING agonist MSA‐2 and the IDO inhibitor NLG‐919 (71). MSA‐2 locally activates the cGAS‐STING pathway in tumors, promoting IFN‐β secretion and dendritic cell maturation, while the synchronously released NLG‐919 inhibits the IDO‐mediated immunosuppressive metabolic pathway and reduces the infiltration of MDSCs. In a Lewis lung cancer model, these PDNPs demonstrated remarkable tumor‐targeted accumulation and retention, with fluorescence signals in the tumor region still increasing at 24 h post‐injection. The PDNPs treatment group exhibited the most significant suppression of tumor volume and a pronounced prolongation of mouse survival. A similar esterase-responsive strategy has also been employed to construct peptide-based prodrugs. Jiang et al. designed an esterase-activated dual-immunomodulatory stapled peptide nanoprodrug (sKLA-NLG NPs), in which the biaryl-stapled KLA peptide (sKLA) with enhanced stability and membrane permeability was covalently conjugated to the IDO inhibitor NLG-919 via an ester bond (**Figure 4**), subsequently self-assembling into nanoparticles driven by amphiphilicity (36). The stapled architecture endowed sKLA with a 4.4-fold longer serum half-life and a 2.5-fold higher cellular uptake compared to its linear counterpart. Within the TME, esterase-mediated hydrolysis of the ester bond triggered the simultaneous release of sKLA and NLG-919. sKLA induced mitochondrial membrane potential depolarization and cytochrome c release, leading to a 2.16-fold increase in CRT exposure and a 4.97-fold enhancement in HMGB1 release. Concurrently, NLG-919 inhibited IDO activity, reducing kynurenine production in tumor cells by 65.9%. When combined with an anti-PD-L1 antibody, this nanoprodrug achieved 88.0% inhibition of primary tumors and 97.3% suppression of distant tumors in an orthotopic 4T1 breast cancer model, along with a significant reduction in lung metastatic nodules.

Beyond the selection of an enzyme target, the location and kinetics of enzymatic cleavage can also critically influence the magnitude and duration of immune activation, making linker design an important determinant of therapeutic performance. Wang et al. designed β-glucuronidase-responsive TLR7/8 agonist prodrug vesicles (72). IMDQ was covalently linked to PEG via a linker containing a β-glucuronidase-sensitive motif (glucuronide) and a self-immolative benzyl carbamate chain, forming an amphiphilic prodrug molecule (PEG-GL2-IMDQ). This molecule self-assembles in water into vesicles approximately 200 nm in diameter. After endocytosis by antigen-presenting cells, the vesicles, within the endosomal acidic environment, are first cleaved by esterases at the ester bond, followed by β-GUS-mediated removal of the glucuronide group. This triggers a self-immolative reaction, releasing native IMDQ, which subsequently activates the TLR7/8 signaling pathway. Following subcutaneous injection in mice, these vesicles induced interferon-β expression at the injection site and in the draining lymph nodes with an intensity more than three times greater than that of the control group, along with a significantly prolonged duration. To precisely achieve tumor-specific rapid activation of TLR7/8 agonist prodrugs, Zhang et al. developed a TLR7/8 agonist prodrug delivery platform based on polypropylene sulfide nanoparticles (73). This platform precisely modulates the kinetics of immune activation by designing linkers with different enzyme-responsive properties. The alanine dipeptide linker in this platform can be rapidly recognized and cleaved by lysosomal proteases such as papain, enabling rapid drug release. Under conditions mimicking the acidic lysosomal environment (pH 5.5) and in the presence of the model protease papain, the Ala-linked prodrug released approximately 30% of the IMDQ agonist within 2 days. In contrast, the Alkyl linker released only about 60% over 10 days under the same acidic conditions and was insensitive to protease. Following a single subcutaneous injection of Ala-linked polypropylene sulfide-IMDQ nanoparticles, a sustained increase in the number of dendritic cells and macrophages in the draining lymph nodes was observed for over 7 days. Nevertheless, the rapid-release mode mediated by the Ala linker induced relatively low levels of IL-12 secretion and failed to elicit the sustained, high-level production of IL-12 induced by the slow-release Alkyl linker.

Collectively, enzyme-responsive PDNPs offer substantial molecular programmability because cleavable linkers can be tailored to enzymes located in different biological compartments. Extracellular enzymes such as MMPs can be exploited to regulate nanoparticle de-shielding, tissue penetration, or component release before cellular uptake, whereas lysosomal enzymes such as CatB and other proteases are more suitable for intracellular prodrug activation. This spatial diversity provides an opportunity to control different stages of the delivery and activation process. However, enzyme responsiveness should not be regarded as equivalent to tumor exclusivity, because both enzyme abundance and catalytic activity may vary across tumors and within different regions of the same lesion, while substrate accessibility is additionally influenced by cellular uptake and subcellular trafficking. Therefore, rational enzyme-responsive PDNP design requires simultaneous consideration of enzyme specificity, spatial accessibility, and cleavage kinetics. Although cascade or multi-enzyme-responsive strategies may further improve activation selectivity, the resulting increase in structural complexity should be balanced against formulation reproducibility and translational feasibility.

### 2.4 Hypoxia-responsive PDNPs

The hypoxic TME serves as a critical pathological feature contributing to therapeutic resistance and immune evasion, and is closely linked to key characteristics of tumor cells such as resistance to cuproptosis and genomic instability (74, 75). Conventional therapeutic agents often suffer from nonspecific distribution and diminished activity under hypoxic conditions, rendering them ineffective in achieving sufficient enrichment and activation within these regions (76, 77). The fundamental design principle of hypoxia-responsive PDNPs lies in utilizing hypoxia, a unique signal within the TME, as a specific trigger. This approach converts the inertness of prodrugs under physiological conditions into selective activation at the disease site, thereby achieving targeted therapy against hypoxic tumor cells.

Hypoxia, a hallmark feature of solid tumors caused by aberrant vasculature and metabolic dysregulation, creates a distinct biochemical niche. This niche is characterized by elevated nitroreductase (NTR) activity and hypoxia-inducible factor-1α (HIF-1α)-mediated enzymatic cascades, factors that can be leveraged for the tumor-selective activation of immunomodulatory agents. Zhou et al. designed a hypoxia-responsive prodrug (JNP) based on a nitroaromatic linker, which covalently conjugates PTX with the BRD4 inhibitor JQ1 (78). In the presence of DSPE-PEG_2000_, JNP self-assembled into nanoparticles (DJNP NPs). Within the hypoxic TME, the nitroaromatic linker is reduced by NTR, triggering a cascade of self-elimination reactions that lead to the specific release of PTX alongside the active form of JQ1, JQ1-COOH. The released PTX induced ICD, significantly promoting CRT exposure on the cell membrane, HMGB1 release, and ATP secretion. This process consequently enhanced dendritic cell maturation and cytotoxic T lymphocyte infiltration. Simultaneously, JQ1 effectively downregulated the chemotherapy-induced overexpression of PD-L1, thereby reversing immunosuppression. In the 4T1 breast cancer model, treatment with the high-dose DJNP NPs group (PTX 30 mg/kg) resulted in a significantly higher number of intratumoral CD3^+^CD8^+^ T cells compared to the Abraxane group. Based on a similar principle, Zhang et al. developed a CPT-based dimeric prodrug (CP), constructed via a nitroaromatic linker bridge (79). This linker can be catalytically reduced by NTRs under hypoxic conditions, initiating an electron cascade and self-immolation reaction to release CPT, which possesses ICD-inducing activity. In a 4T1 breast cancer model, the CPPA nanomicelles significantly enhanced intratumoral drug accumulation and achieved over 80% drug release under hypoxic conditions. This therapy not only effectively suppressed the growth of orthotopic tumors but also markedly reduced the number of lung metastatic foci, demonstrating excellent anti-metastatic capability. To further refine the release mechanism of the prodrug system, subsequent studies have introduced more precise activation strategies. Another study reported a hypoxia/NTR-responsive diethyldithiocarbamate (DTC) prodrug (N-DTC), which was co-loaded with a curcumin-Cu²^3^ complex (Cur-Cu) into bovine serum albumin-modified nanoparticles (CCND@BSA) (**Figure 5**), constructing a dual-prodrug activation system based on ligand competition (80). The cytotoxicity of CCND@BSA against 4T1 cells was significantly stronger under hypoxic conditions than under normoxic conditions, reducing cell viability to 10.3 ± 1.2% at a concentration of 320 μg/mL. Meanwhile, it induced a late-stage apoptotic cell proportion of up to 55.9%, and significantly enhanced CRT exposure and HMGB1 release. A further strategy to enhance the efficacy of hypoxia-activated prodrugs involves actively modulating the TME. Xu et al. developed the urea-modified tirapazamine derivative TPZP, which retains the hypoxic activation core structure of TPZ (81). Under tumor hypoxia, TPZP undergoes one-electron reduction to generate radical species that cause DNA damage and thereby exert cytotoxic effects. The study further fabricated TPZP into a fibrin-targeting nanodrug, FT11-TPZP-NPs. In a combination regimen with the vascular disrupting agent CA4-NPs, CA4-NPs increased intratumoral fibrin expression by approximately 9-fold and up-regulated the hypoxia marker HIF-1α by 2.2-fold, thereby significantly promoting the accumulation and activation of TPZP within tumors. In a CT26 colon carcinoma model, a single dose of FT11-TPZP-NPs combined with CA4-NPs achieved a tumor inhibition rate of 98.1%, with complete regression observed in four out of six tumors.

**Figure 5:**
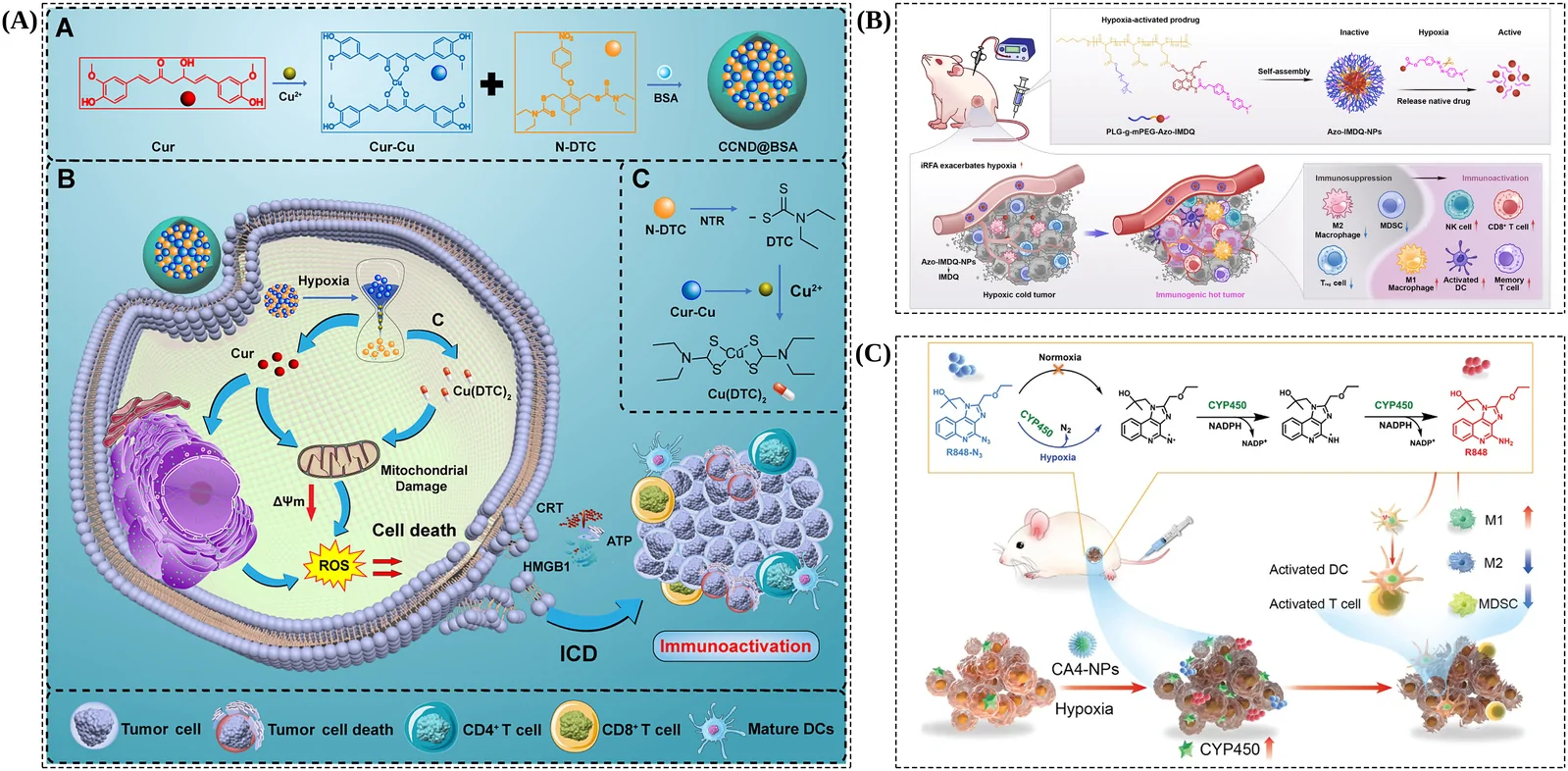
**(A)** Preparation of CCND@BSA and schematic representation of the ligand-competition strategy-based dual prodrug activation mechanism for synergistic therapy (80). Copyright 2026, Elsevier **(B)** Schematic illustration of the fabrication of hypoxia-activated Azo-IMDQ-NPs and the mechanism of released IMDQ to potentiate iRFA mediated antitumor immunity through reprogramming the “cold” tumor immune microenvironment (82). Copyright 2025, Wiley **(C)** Schematic diagram showing the selective tumor therapy of R848-N3. R848-N3 is only reduced by the CYP450 enzyme in a hypoxic tumor environment. Under hypoxic conditions, CYP450 enzyme catalyzes R848-N3 to produce N2 (non-toxic), and the resulting radicals further carry out irreversible sequential single-electron transfer. Then the reduction of NADPH leads to the formation of R848, thereby achieving the selective activation of R848-N3. R848-N3 is only activated in hypoxic areas of the tumor tissue to provide a selective therapy, and tumor selectivity can be achieved by increasing the level of tumor hypoxia (85). Copyright 2023, Wiley.

The azobenzene (Azo) structure can be cleaved by azoreductase under hypoxic conditions, enabling controlled drug release. In recent years, hypoxia-responsive prodrug nano-systems based on Azo linkages have been extensively developed to achieve localized release of immunomodulators in tumors and reduce systemic toxicity. In a recent study, Zhang et al. developed a copper ion competition-based nano-exchanger (PADSC NPs) (42). This nanosystem consists of an Azo-linked DTC precursor and a copper-chelated fluorescence quencher SQ-Cu. Under hypoxic conditions, the Azo linker is cleaved by overexpressed azoreductase within the tumor, releasing DTC. DTC then undergoes Cu²^3^ competitive exchange with SQ-Cu, with a binding constant as high as 6.37×10^4^ M^-^¹, leading to the *in situ* generation of DTC-Cu complexes with ICD-inducing activity. This process is accompanied by the recovery of SQ fluorescence, enabling visualization of the treatment. In a 4T1 tumor-bearing mouse model, PADSC NPs achieved a 79.23% inhibition of tumor growth, induced intratumoral CD8^+^ T cell infiltration of 34.5% ± 0.4%, and elevated serum levels of TNF-α and IFN-γ to 4.55-fold and 2.77-fold of the control group, respectively. However, relying solely on endogenous hypoxia levels within tumors limits the activation efficiency due to spatial heterogeneity in the degree of tumor hypoxia. To overcome the insufficient activation by endogenous hypoxia, Ji et al. constructed Azo-linked IMDQ PDNPs (Azo-IMDQ-NPs), which combine a hypoxia-responsive strategy with local thermal ablation therapy to actively aggravate tumor hypoxia (82). *In vitro*, Azo-IMDQ-NPs were selectively and nearly completely (≈100%) reduced under severely hypoxic conditions mimicking the post-iRFA microenvironment, releasing the TLR7/8 agonist IMDQ (**Figure 5**). In a subcutaneous H22 hepatocellular carcinoma model, the combination of iRFA and Azo-IMDQ-NPs achieved a tumor suppression rate of 96.6%, with over 80% of mice surviving beyond 60 days, and successfully inhibited contralateral tumor growth. This therapy increased the intratumoral proportion of M1 macrophages from 1.75% to 4.17% and elevated cytotoxic CD8^+^ T cell infiltration by 6.34-fold, demonstrating its ability to actively leverage therapy-aggravated hypoxia to elicit potent systemic antitumor immunity. To further augment the immunotherapeutic synergy and directly remodel the immunosuppressive TME, Huang et al. devised a multimodal strategy involving the design of hypoxia-responsive albumin-based nanoparticles (HACS NPs) co-delivering the hypoxia-activated ICD inducer AQ4N, the STING agonist SR-717, and CaCO_3_ (83). This system employs an Azo-based crosslinker, which dissociates in the hypoxia-aggravated environment post-HIFU treatment, enabling the synchronous release of the encapsulated components. Under hypoxic conditions, the release rate of the key component AQ4N reached 92.21%. Specifically, AQ4N is reduced to its toxic form, AQ4, in hypoxia, effectively inducing ICD, as demonstrated by a significant increase in cell surface CRT exposure and HMGB1 release. On another front, SR-717 activates the STING pathway, promoting the production of type I interferons, which drove the maturation rate of bone marrow-derived dendritic cells to approximately 70%. Furthermore, CaCO_3_ efficiently depletes lactate, thereby reprogramming the TME and reversing the M1/M2 macrophage ratio from a baseline favoring the immunosuppressive M2 phenotype to a pro-inflammatory ratio of 3.88 (M1/M2) following HACS NPs + HIFU treatment.

A hypoxia-selective activation system based on azido reduction leverages cytochrome P450 enzymes (CYP450), which are active under hypoxic conditions, to reduce the azido group (–N_3_) to an amino group, thereby activating the prodrug. Xu et al. first employed the vascular disrupting agent CA4-NPs to remodel the TME, inducing vascular occlusion and exacerbating hypoxia (84). Subsequently, a nanoprodrug surface-decorated with a peptide targeting coagulation sites (apictide), namely apictide-PLG-IMDQ-N_3_, was administered. This prodrug is specifically activated under hypoxia via CYP450-mediated reduction of its azido group (–N_3_) to an active amino group, releasing IMDQ. In tumors pretreated with CA4-NPs, the accumulation of activated IMDQ reached 22.66%ID/g. Compared to direct injection of the small-molecule prodrug IMDQ-N_3_, the total amount of activated IMDQ in tumors was enhanced approximately 59.69-fold. In solid tumor models of 4T1 and CT26 with a volume of ≈500 mm³, this sequential therapy achieved complete tumor regression without recurrence within 60 days, demonstrating strong curative potential. In terms of immunotherapy, the treatment significantly increased the proportions of intratumoral cytotoxic CD8^+^ T cells and M1-like macrophages, and induced the generation of central memory and effector memory T cells in the spleen, establishing long-term immunological memory. Although the aforementioned sequential system showed significant efficacy, the activation of its prodrug relied on a two-step process of ester bond hydrolysis and azide reduction, the efficiency of which might be complicated by the intricate enzymatic activity within the TME. A simpler strategy was adopted by directly masking the active amino group of R848 (another TLR7/8 agonist) with an azide moiety, yielding the R848-N_3_ prodrug (85) (**Figure 5**). This prodrug can be activated via a single key step: hypoxia-dependent CYP450 reduction. R848-N_3_ exhibited minimal systemic toxicity. At an equivalent dose, the serum levels of pro-inflammatory cytokines IL-6 and IFN-γ it induced were merely 1/12 and 1/16 of those elicited by free R848, respectively. Concurrently, its maximum tolerated dose in mice far exceeded that of R848. When combined with CA4-NPs to further intensify tumor hypoxia, R848-N_3_ demonstrated exceptionally selective activation at the tumor site. At 1hour post-administration, the concentration of active R848 in the tumor was 21.45-fold higher than that in the heart. Furthermore, in a more clinically relevant combination therapy experiment, the regimen of R848-N_3_ plus CA4-NPs and anti-PD-1 antibody achieved a 94.1% tumor suppression rate and realized a 40% complete cure rate in the 4T1 breast cancer model, with an impressive 80% survival rate at day 60 in the treatment group.

Overall, hypoxia-responsive PDNPs are particularly attractive because they can convert a pathological feature associated with therapeutic resistance into a trigger for localized prodrug activation. The systems discussed above illustrate several complementary chemical strategies, including nitroaromatic reduction, azo cleavage, and azide reduction, and further demonstrate that hypoxia can be actively intensified by vascular disruption or local ablation to amplify prodrug activation. Nevertheless, hypoxia is intrinsically heterogeneous and dynamic, making the extent of activation difficult to predict when a formulation relies solely on pre-existing tumor hypoxia. Therapeutically induced hypoxia can partially overcome this limitation, but it also introduces additional dependence on treatment sequence, local intervention, and the reproducibility of microenvironmental remodeling. Moreover, the comparison between multistep and directly masked prodrugs suggests that increasing activation complexity is not always necessary; simplified activation chemistry may reduce dependence on multiple biological processes while retaining tumor selectivity. Therefore, the design of hypoxia-responsive PDNPs should balance activation sensitivity with chemical simplicity and should distinguish whether hypoxia serves as a pre-existing tumor biomarker or as a therapeutically generated trigger.

## 3 Exogenous-activatable PDNPs

Although endogenous physiological stimuli offer a convenient strategy for spatial control in cancer immunotherapy, they lack the potential for precise temporal control. In particular, once therapeutic agents reach tumor cells or the TME, endogenous stimulation will promptly trigger the release of the therapeutic payload from PDNPs (86). However, employing nanoplatforms responsive to exogenous stimuli can facilitate the development of cancer immunotherapies with higher spatiotemporal precision. Among the currently applied stimuli, light serves as a safe modality for controlled drug delivery due to its unparalleled advantages, such as high precision and minimally invasive nature (87, 88). By adjusting the irradiance, duration, and location of the light source, precise control over the functionality of light‐controlled drug delivery systems can be achieved (89, 90). In addition to light response, ultrasound also serves as a promising modality for activating exogenous nanoprodrugs. The cavitation effect of ultrasound enhances the permeability of tumor blood vessels and cells, and by modulating the intratumoral and intracellular structure and pharmacokinetic behavior of PDNPs, it can potentiate the antitumor therapeutic efficacy (91, 92).

### 3.1 Light-responsive PDNPs

Traditional activation strategies for photo-responsive PDNPs predominantly rely on visible/ultraviolet light-responsive systems, which enhance the efficacy of immunotherapy by triggering auxiliary drug release or modulating the TME upon light irradiation. For instance, Choi et al. designed visible-light-triggered PDNPs activated by 671 nm visible light (93). Their structure is based on a cathepsin B-cleavable peptide (FRRG) linking the photosensitizer VPF and the chemotherapeutic drug DOX. The overexpressed CatB in tumor cells enables specific drug release. Under light irradiation, this system simultaneously generates ROS and exerts chemotherapeutic effects, synergistically inducing ICD. Notably, monotherapy with visible-light-triggered PDNPs (+L) achieved a 60% complete response rate in a colon cancer model. Furthermore, when combined with PD-L1 blockade, visible-light-triggered PDNPs (+L) mediated a 100% complete response. This combinatory treatment significantly increased the proportion of intratumoral CD8^+^ T cells to 41.1%, substantially reduced the populations of immunosuppressive cells such as regulatory T cells and MDSCs, and successfully established long-term anti-tumor immunological memory. In addition to directly acting on tumors, light-responsive ROS can also activate prodrugs. One study designed a ROS/photo dual-responsive prodrug unimolecular micelle PPTN (41). This system utilizes 630 nm light excitation, links the photosensitizer protoporphyrin IX with the IDO inhibitor NLG-919 via a thioketal bond, and introduces a PEG chain to form a unimolecular micelle. Under light irradiation, the ROS generated by photosensitizer protoporphyrin IX not only directly kills tumor cells and induces ICD but also cleaves the thioketal bond, enabling controlled release of NLG-919, thereby reversing IDO-mediated immunosuppression (**Figure 6**). In a bilateral 4T1 tumor model, PPTN combined with light irradiation significantly enhanced the growth inhibition of distant metastatic tumors, increased the proportion of CD8^+^ T cells within the tumors, markedly elevated serum IFN-γ levels, and did not cause significant systemic toxicity. However, due to the extremely limited tissue penetration capacity of visible and ultraviolet light, it is difficult to act on deep-seated or large solid tumors, which severely restricts its clinical translation prospects.

**Figure 6:**
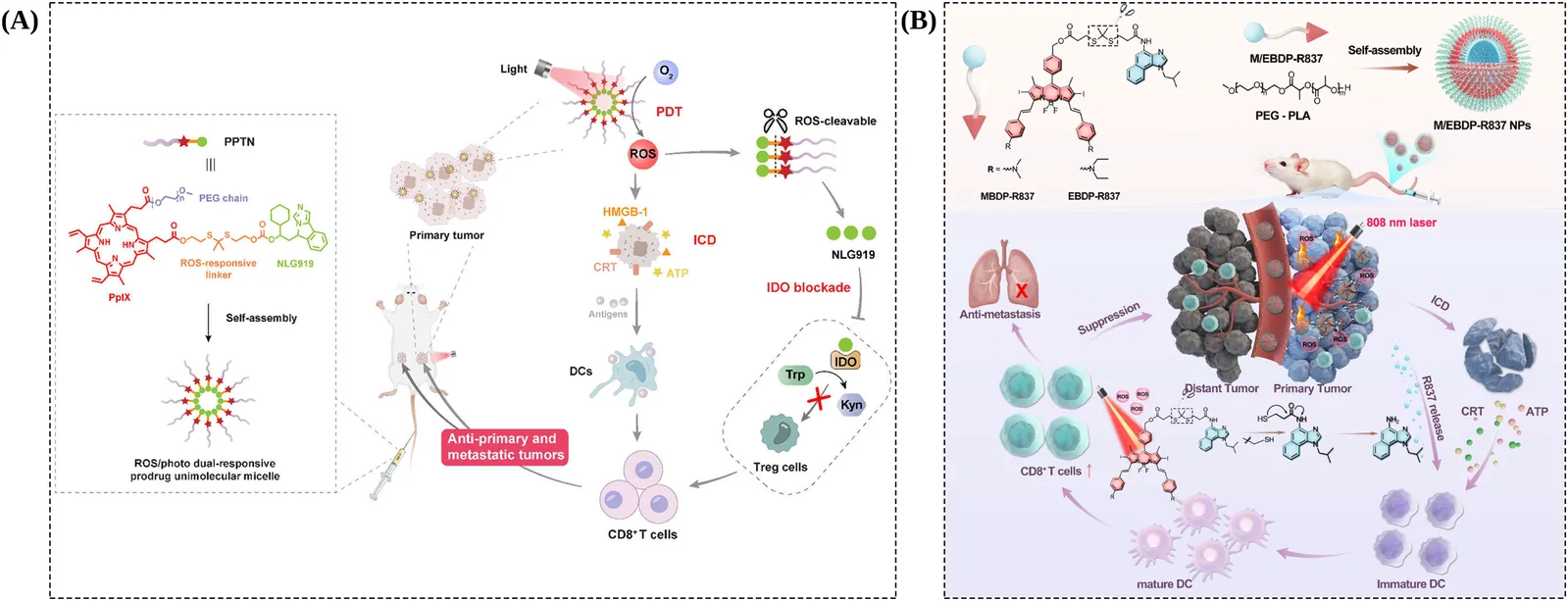
**(A)** Schematic illustration of the construction of the ROS/photo dual-responsive prodrug unimolecular micelle PPTN and the *in vivo* therapeutic mechanism of PPTN for boosted cancer photodynamic immunotherapy (41). This image is available under the CC BY 4.0 license. **(B)** Near-infrared light-activated immunomodulator prodrugs for photoimmunotherapy of “cold” triple-negative breast cancer (94). Copyright 2025, Wiley.

More advanced strategies leverage near-infrared light-responsive systems by extending the excitation wavelength into the region with greater tissue penetration, offering a viable pathway for precise immunotherapy of deep-seated tumors. For instance, Shen et al. reported a class of 808 nm light-activatable immunomodulator prodrugs (M/EBDP-R837) (94). The core mechanism lies in utilizing the near‐infrared-excitable BODIPY photosensitizer to generate ROS, which specifically cleave the thioketal linker, thereby enabling spatiotemporally controlled release of the TLR7 agonist R837 at the tumor site (**Figure 6**). In the 4T1 triple-negative breast cancer model, this system achieved a 67.1% release rate of R837 under 808 nm laser irradiation. It concurrently induced significant ICD and dendritic cell maturation. Ultimately, the treatment led to complete cure in 83% of the mice, with no observed lung metastasis. Based on a similar principle, a hypoxia-tolerant polymeric photosensitizer prodrug, HTPsNiclo, was further developed (95). It also utilizes 808 nm excitation but operates via a Type-I photodynamic mechanism to continuously generate O_2_•^-^ and •OH even under hypoxic conditions. Additionally, it leverages the overexpression of CatB in the TME to trigger the release of the STAT3 inhibitor Niclosamide. *In vitro*, HTPsNiclo achieved 96.47% release of Niclosamide upon incubation with CatB, downregulated HIF-1α expression, and significantly enhanced immune cell activation. In the 4T1 tumor model, HTPsNiclo-mediated photo-immunotherapy enhanced the tumor inhibition rate to 91.23%, compared to 75.53% for Type-I PDT alone. It also prolonged the 100% survival rate from 39 days (Type-I PDT alone) to 60 days, while effectively inhibiting the growth of distant tumors and lung metastasis.

Furthermore, current research has moved beyond simple light-triggered release and is now focusing on leveraging the deep‐tissue penetration advantage of near‐infrared light in combination with more sophisticated response logic, to construct cascade‐responsive and combination‐therapy platforms capable of overcoming tumor heterogeneity. A recent study designed a light‐triggered PROTAC nanoassembly (LPN) (96). This system covalently links the photosensitizer VPF to an IDO‐targeting PROTAC via a CatB‐responsive peptide (KRR), forming nanoparticles that self‐assemble in aqueous solution. In the absence of light, PROTAC activity remains “silenced” to avoid systemic off‐target degradation. At the tumor site, CatB overexpression cleaves the peptide linkage, releasing active PROTAC and VPF. Subsequent near-infrared irradiation triggers ROS generation by VPF, inducing ICD in tumor cells while enabling continuous IDO proteolysis. These dual mechanisms work synergistically to reverse the immunosuppressive TME. The LPN produced ROS levels significantly higher than those in the control group under light irradiation and markedly downregulated both IDO expression and the Kyn/Trp ratio. In CT26 colon carcinoma models, the LPN + light group achieved 100% complete regression, effectively suppressed lung metastasis, and established immunological memory. For deeper-seated or diffuse lesions, Zhang and colleagues further utilized a selenium-modified organic chromophore (TTBSe) as a near-infrared-II photothermal and photoacoustic dual-function probe (97). Upon 1064 nm laser irradiation, this probe enabled high-contrast near-infrared-II photoacoustic imaging while concurrently triggering a mild photothermal effect and activating the thermo-responsive prodrug PTX-Azo to release the chemotherapeutic agent. In an orthotopic GL261 glioma model, the nanoplatform (SeP@MT NPs), leveraging the dual-targeting strategy of a biomimetic membrane and the T7 peptide, enhanced blood-brain barrier penetration and tumor accumulation by approximately 2.2-fold compared to the non-targeted control. Under near-infrared-II light irradiation, through the synergistic combination of photothermal therapy, chemotherapy, and immunotherapy, it achieved a 91.5% tumor suppression rate.

Taken together, light-responsive PDNPs provide a distinct advantage over purely endogenous stimulus-responsive systems by allowing drug activation to be externally controlled in both space and time. The progression from visible-light-responsive systems to NIR-I and NIR-II platforms has partially addressed the limitation of tissue penetration while simultaneously enabling the integration of photodynamic, photothermal, and imaging functions. In addition, combining light activation with enzyme-responsive cleavage or active targeting can further separate tumor localization from subsequent drug activation, thereby improving multistage control. Nevertheless, improved optical penetration does not eliminate the requirement for adequate light delivery to the entire target lesion. Treatment efficacy may therefore remain dependent on tumor depth, anatomical accessibility, irradiation geometry, and the ability to achieve sufficiently uniform energy deposition throughout heterogeneous or diffuse tumors. Furthermore, increasingly multifunctional photoresponsive systems may require additional photosensitizers, targeting components, or responsive linkers, creating a trade-off between enhanced spatiotemporal precision and formulation complexity. Thus, the translational potential of light-responsive PDNPs should be evaluated not only according to activation efficiency, but also according to whether clinically practical irradiation can reproducibly activate the prodrug throughout the intended tumor volume.

### 3.2 Ultrasound-responsive PDNPs

Utilizing reactive ROS generated by the sonodynamic effect to trigger drug release is a common strategy for constructing ultrasound-responsive nanoscale prodrugs. In a study targeting glioblastoma, Kang et al. developed an ultrasound-responsive nanoplatform named SeM@GL NPs (98). This platform employs the selenium-containing sonosensitizer TB-Se to efficiently produce ROS under ultrasound irradiation (1 MHz, 1.0 W/cm²). The Se NPs (nanoparticles formulated from TB-Se) demonstrated superior sonodynamic performance, causing a 40% decrease in the absorbance of the singlet oxygen indicator DPBF within 5 minutes, whereas the clinically used agent methylene blue showed only a 13% decrease under the same conditions. The generated ROS not only directly induce ICD in tumor cells but also specifically trigger the hydrolysis of the diselenide bond-linked STING agonist prodrug, di-MSA-2, releasing the active STING agonist MSA-2. This activation stimulates the cGAS-STING pathway and promotes IFN-β secretion, with the highest level observed in the SeM@GL NPs + US treatment group. Concurrently, the CXCR4-targeting peptides conjugated on the nanoparticle surface block the CXCL12/CXCR4 axis. In an orthotopic Glioblastoma mouse model, this combination therapy significantly inhibited the infiltration of immunosuppressive cells, reducing the intratumoral proportion of MDSCs to 7.7% in the SeM@GL NPs + US group. Similarly, Song et al. designed a piezoelectric nanoparticle system, BTO@BAL, based on the piezocatalytic effect of barium titanate (99). Under ultrasound irradiation (1 MHz, 1.5 W/cm²), the system converted mechanical energy into chemical energy, generating ROS (**Figure 7**). The produced ROS oxidized (Arg)9 to release nitric oxide, which reduced tumor stiffness from ~24.68 kPa to ~14.39 kPa post-treatment. Concurrently, ROS cleaved the thioketal bond, triggering the release of the PD-L1 inhibitor BMS1166 and downregulating PD-L1 expression on tumor cells by approximately 42.9%.

**Figure 7:**
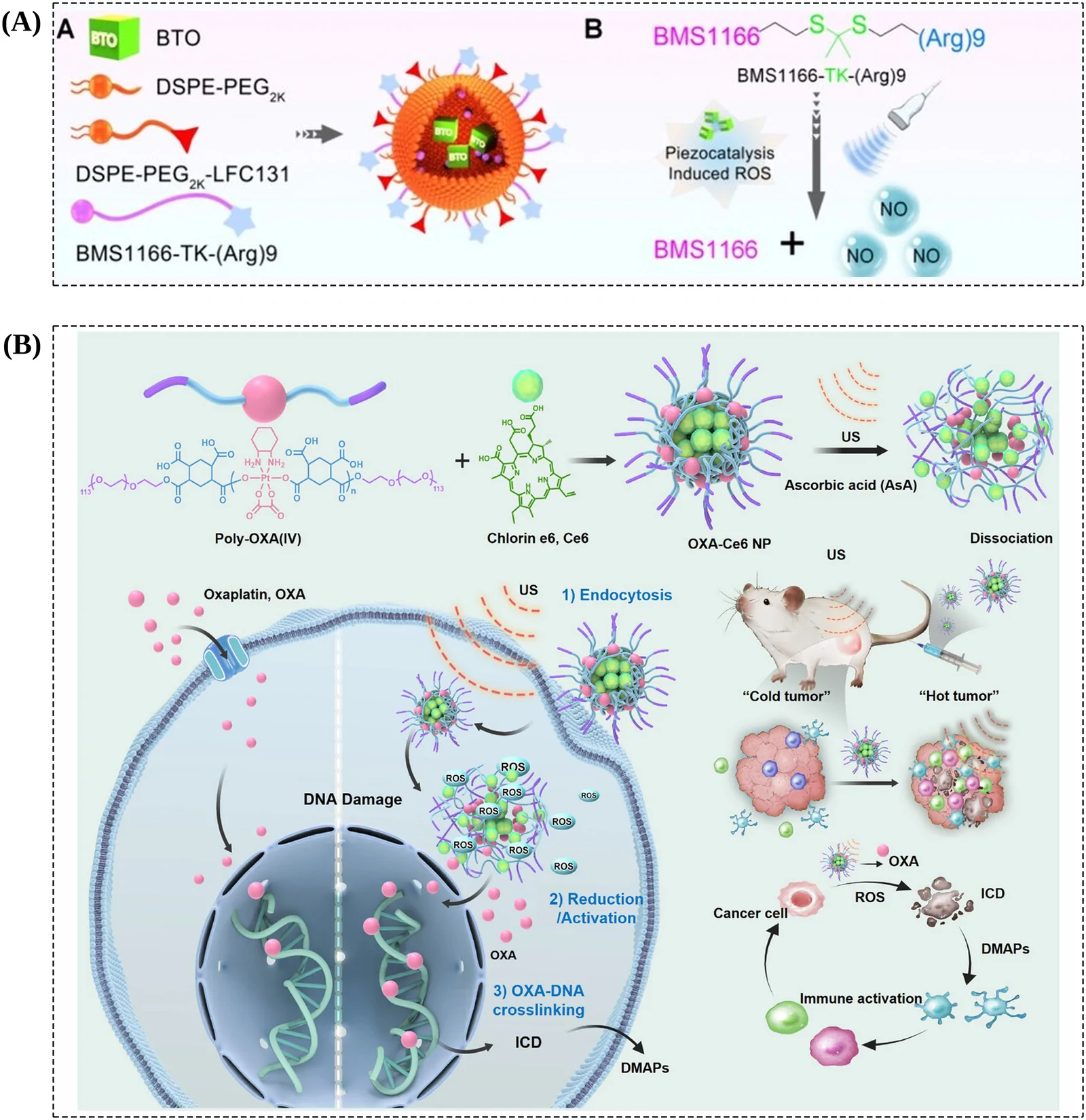
**(A)** Schematic illustration of the preparation of BTO@BAL nanoparticles, the chemical structure and release mechanism of the prodrug molecule (99). Copyright 2025, American Chemical Society. **(B)** Schematic illustration of the US-triggered cascade delivery system for Poly-OXA nanoparticles. Poly-OXA(IV) was co-assembled with a sonosensitizer Ce6 to form OXA-Ce6 NP, characterized by robust sono-responsiveness (101). Copyright 2025, Wiley.

Beyond ROS-mediated bond cleavage and piezocatalysis, ultrasound can also initiate reductive chemical transformations for direct prodrug activation. For instance, a representative study addressed the issue of off-target activation associated with the clinical TLR7/8 agonist IMDQ (100). By designing an azide-modified nanoprodrug (IMDQ-N_3_ NPs), the active amino group (-NH_2_) of IMDQ, which is essential for TLR7/8 binding, was replaced with an azide group (-N_3_) serving as a “chemical masking moiety”. This enabled spatiotemporally selective immune activation triggered by ultrasound. Co-delivery of the prodrug with the sonosensitizer riboflavin within a nanocarrier constructed a “dormant” drug delivery system capable of specific ultrasound-activated drug release. Upon ultrasound irradiation at the tumor site, the sonosensitizer significantly enhanced the cavitation effect, promoting water molecule cleavage to generate a large number of hydrogen radicals. These radicals efficiently reduced the inert azide group back to the active amino group, thereby restoring the drug’s immunostimulatory function. In tumor tissue homogenate, the drug activation efficiency of these nanoparticles under ultrasound was 12.2-fold higher than that of the control group without the sonosensitizer. In a CT26 colon cancer mouse model, following ultrasound irradiation, the concentration of the active drug within the tumor was 35.2-fold higher than that in the non-irradiated free prodrug group, with a tumor-to-normal tissue (e.g., heart) drug concentration ratio as high as 196.3:1. The precision activation achieved thereby allowed the treatment regimen to attain a 95.7% tumor suppression rate and a 60% long-term survival rate, while completely avoiding the body weight loss and systemic cytokine storm induced by conventional IMDQ therapy, resulting in fundamental control over systemic toxicity. To enhance drug delivery into tumor cells and overcome deep-seated resistance barriers, a more sophisticated strategy was employed. This involved integrating an amphiphilic polymer incorporating the oxaliplatin (IV) prodrug (OXA(IV)) with the sonosensitizer Ce6 through co-assembly, constructing ultrasound-responsive poly-oxaliplatin nanoparticles (OXA-Ce6 NP) (101) (**Figure 7**). Upon ultrasound exposure, this system achieved cascade drug delivery: cellular uptake increased by ~1.7-fold, followed by Ce6-mediated reduction and release of active OXA(II) (85% within 48 h). Finally, the ultrasound microenvironment significantly promoted the cross-linking of OXA with tumor cell DNA, elevating the formation of platinum-DNA adducts, a key DNA damage marker, to approximately 4.8 times the level observed with OXA treatment alone. This cascade-amplified mechanism not only reduced the IC50 to 1.18 µM in Hepa1–6 cells but also potently induced ICD. Post-treatment analysis revealed a significant increase in CRT exposure on the tumor cell surface and a marked augmentation in ATP release. A substantial proportion of dendritic cells underwent maturation. In a CT26 tumor-bearing mouse model, this strategy demonstrated robust tumor growth inhibition. Moreover, when combined with anti-PD-1 antibody therapy, it further remodeled the tumor immune microenvironment, induced a population of approximately 20% central memory T cells in the spleen, and established durable anti-tumor immune memory.

Overall, ultrasound-responsive PDNPs provide externally controlled activation while offering a broader range of activation mechanisms than simple stimulus-induced bond cleavage. As illustrated by the systems discussed above, ultrasound can generate ROS through sonodynamic or piezocatalytic processes, promote reductive chemical conversion through cavitation-associated radical generation, and facilitate multistage drug delivery and activation. These features make ultrasound particularly attractive for combining local prodrug activation with ICD induction and immune modulation. Compared with light-responsive systems, ultrasound also offers greater potential for treating less superficially accessible lesions. Nevertheless, the reproducibility of ultrasound-triggered activation depends not only on the chemical design of the prodrug but also on physical treatment parameters and the efficiency with which acoustic energy is converted into the intended chemical event. Differences in sonosensitizers, ultrasound intensity, exposure conditions, and activation mechanisms may therefore complicate cross-platform comparison and optimization. In addition, incorporating sonosensitizers, piezoelectric materials, targeting ligands, and multiple responsive components can substantially increase formulation complexity. Thus, future ultrasound-responsive PDNP design should prioritize a clear relationship between acoustic input, prodrug activation, and therapeutic output, while minimizing unnecessary structural complexity to facilitate reproducible and clinically controllable treatment.

## 4 Active targeting and hybrid strategies for PDNPs

In addition to regulating prodrug activation through endogenous or exogenous stimuli, tumor selectivity can be further improved by controlling the localization and cellular recognition of PDNPs. Active targeting provides such an additional level of regulation by introducing ligands, antibodies, peptides, or biomimetic interfaces that preferentially interact with tumor-associated molecular or cellular targets. Importantly, active targeting and stimulus-responsive activation regulate different stages of the delivery process: the former primarily determines where and with which cells the nanoplatform preferentially interacts, whereas the latter governs when and under what conditions the masked therapeutic component is converted into its active form. These two strategies are therefore complementary rather than mutually exclusive and can be integrated to achieve multistage control over PDNP delivery and activation.

Active targeting can be incorporated into PDNPs through antibodies, peptides, or biomimetic interfaces to improve tumor or cell-specific localization before prodrug activation. In mAb-CD163-PDNPs, a CD163^+^ monoclonal antibody directs the nanoparticles toward CD163-positive tumor-associated macrophages, while intracellular DOX release is controlled by acid-sensitive Schiff-base hydrolysis (59). In hypoxia-responsive systems, FT11-TPZP-NPs exploit fibrin targeting to enhance tumor localization following vascular disruption, whereas apictide-PLG-IMDQ-N_3_ targets coagulation sites before hypoxia-dependent azide reduction activates IMDQ (81, 84). A similar strategy has also been applied to externally triggered systems. SeP@MT combines a biomimetic membrane with the T7 peptide to facilitate blood–brain barrier penetration and glioma accumulation, followed by NIR-II-triggered activation of the thermo-responsive prodrug (97). Cancer-cell-membrane-coated CM@ER-Rh NPs exploit homologous recognition to enhance tumor accumulation, followed by intracellular acidic dissociation and release of the active Rh(I) complex (102). Similarly, the full-API DIM@M platform combines tumor-cell-membrane-mediated homologous targeting with TME-responsive drug release and NIR-triggered therapeutic activation (103). In these systems, active recognition primarily improves the spatial distribution of the nanoplatform, while prodrug activation remains controlled by the corresponding endogenous or exogenous stimulus.

More complex PDNP designs can combine several responsive processes within a single therapeutic sequence to improve the control of drug activation. One example is the cascade pH-responsive PDNP, which first undergoes dePEGylation and size reduction in the mildly acidic extracellular tumor environment, followed by further disassembly and drug release in the more acidic intracellular compartment (58). Logic-gated systems provide another approach. An “AND” logic-gated prodrug micelle requires two tumor-associated inputs for efficient activation, markedly reducing drug release when only one stimulus is present (62). External treatments can also be used to modify the local microenvironment before prodrug activation. For instance, iRFA aggravates tumor hypoxia and thereby promotes the reduction of Azo-IMDQ-NPs and subsequent IMDQ release (82), whereas HIFU-induced hypoxia facilitates the dissociation of Azo-crosslinked HACS NPs and the release of hypoxia-activated therapeutic components (83). Such sequential designs allow one biological or physical event to create favorable conditions for the next stage of delivery or activation, thereby providing additional control beyond a single stimulus-responsive process.

Endogenous, exogenous, and active-targeting strategies provide different levels of control over PDNP delivery and activation. Endogenous stimulus-responsive systems can exploit pathological features of the TME without additional intervention, but their activation efficiency may vary with the spatial and temporal heterogeneity of tumor-associated signals. Exogenous stimuli offer greater temporal control and allow treatment to be initiated at a defined location, although their performance remains dependent on the penetration and distribution of the applied physical energy within the tumor. Active targeting can further improve the preferential localization or cellular uptake of PDNPs by directing them toward selected molecular or cellular targets, but this process primarily governs delivery rather than prodrug activation itself. Combining these strategies may therefore provide more stringent control by separating tumor localization from subsequent drug activation. However, increasingly complex designs also introduce additional structural components and response steps, which may compromise formulation simplicity and reproducibility. The choice of strategy should therefore be guided by whether each additional targeting or responsive element provides a meaningful improvement in therapeutic selectivity rather than by maximizing the number of functional components within a single platform.

## 5 Clinical translation and future perspectives

### 5.1 Clinical lessons relevant to PDNP development

Clinical experience with conventional IDO-pathway inhibitors and innate immune agonists provides important lessons for PDNP development, particularly regarding therapeutic windows, intratumoral target modulation, systemic exposure, and patient selection. The limited efficacy of immunotherapy in many solid tumors is partly attributable to the immunosuppressive tumor microenvironment (43). Within this context, IDO1 expression can be induced by IFN-γ secreted by activated T cells. IDO1-mediated tryptophan catabolism contributes to local immunosuppression and has been implicated in resistance to immune checkpoint blockade (104). IDO-pathway inhibition has been investigated across multiple tumor settings. Clinically, indoximod has been evaluated in metastatic solid tumors, invasive breast cancer, and pancreatic cancer (105, 106) (**Table 2**). Complementary studies have further highlighted the biological and therapeutic relevance of IDO1 in metastatic melanoma (107) and in preclinical liver cancer models (108). Notably, ECHO-301/KEYNOTE-252 failed to demonstrate an improvement in progression-free or overall survival. Following this negative result, several subsequent phase III epacadostat combination programs were discontinued or curtailed. One proposed explanation for the limited efficacy is that the selected dosing regimen may not have achieved sufficiently sustained intratumoral target inhibition (109). By contrast, indoximod has been explored in combination with cytotoxic therapies rather than relying solely on checkpoint-blockade combinations. It demonstrated manageable safety and encouraging preliminary efficacy signals in early trials for recurrent brain tumors in children (NCT02502708) and newly diagnosed acute myeloid leukemia (NCT02835729) (110, 111). Overall, the clinical development of IDO-pathway inhibitors appears to be shifting from simple combination strategies toward more rational therapeutic combinations, optimized dosing, and biomarker-guided patient selection. From a PDNP design perspective, these clinical outcomes suggest that pathway inhibition alone may be insufficient unless sustained target modulation can be achieved within the tumor while unnecessary systemic exposure is minimized.

**Table 2:** Examples of IDO inhibitors in clinical trials.

IDO inhibitors	Phase	Indication	NCT Identifier	Date	Status
Indoximod	I	Pediatric Brain Tumors	NCT02502708	2015	Completed
	I	Acute Myeloid Leukemia	NCT02835729	2016	Completed
	I/II	Metastatic Breast Cancer	NCT01042535	2009	Completed
	I/II	Metastatic Pancreatic Cancer	NCT02077881	2014	Completed
Epacadostat	I	Advanced Malignancy	NCT03217669	2018	Active, not recruiting
	II	Myelodysplastic Syndromes	NCT01822691	2013	Completed
	III	Melanoma	NCT02752074	2016	Completed
	III	Head and Neck Squamous Cell Carcinoma	NCT03358472	2017	Completed
	III	Urothelial Carcinoma	NCT03374488	2017	Completed
Linrodostat	I/IIa	Advanced malignant tumors	NCT02658890	2016	Completed
	II	Endometrial Cancer or Endometrial Carcinosarcoma	NCT04106414	2019	Active, not recruiting

A related set of translational challenges is encountered with STING and TLR7/8 agonists, which activate innate immunity by promoting dendritic-cell activation and tumor-antigen cross-presentation (112). Although STING and TLR7/8 agonists have demonstrated great potential in enhancing anti-tumor immunity by bridging innate and adaptive immunity in preclinical models, their clinical translation also faces significant challenges (**Table 3**). Two recurring challenges are the risk of systemic inflammatory toxicity when exposure is insufficiently confined and the difficulty of translating strong preclinical immune activation into durable clinical responses (113). Early clinical studies of STING agonists have generally shown limited antitumor activity. The subsequent clinical development of IMSA101 similarly demonstrated limited efficacy: no objective responses were observed with monotherapy in the phase I study, whereas combination therapy with PD-1 blockade produced only a partial response in one patient with uveal melanoma (114). Clinical evaluation of ulevostinag (MK-1454) further showed frequent treatment-related adverse events, including fever and injection-site reactions, while only limited pharmacodynamic evidence of enhanced T-cell infiltration and no substantial tumor regression were observed (115). Collectively, these findings suggest that activation of the STING pathway alone, or its simple combination with checkpoint blockade, may be insufficient to overcome the multiple immunosuppressive barriers present in advanced tumors. TLR7/8 agonists face a related delivery challenge because insufficient tumor selectivity can result in systemic immune activation. To mitigate this risk, intratumoral administration has been explored as a strategy to enhance local exposure while limiting systemic toxicity. For instance, the sustained-release formulation BM201 achieved controlled systemic exposure following intratumoral administration and showed an acceptable early safety profile, although objective responses remained limited (116). Furthermore, the combined antigen release strategies (such as combined vascular blockers or nanodelivery systems) have shown potential to enhance the remote effect in preclinical models, but their clinical validation is still pending (117, 118). These observations highlight a central delivery dilemma for potent innate immune agonists: sufficient local activation is required to initiate effective antitumor immunity, whereas poorly confined exposure can narrow the therapeutic window through systemic immune stimulation.

**Table 3:** Examples of STING agonists in clinical trials.

STING agonists	Phase	Indication	Route of Administration	NCT Identifier	Date	Status
MK-1454	I	Advanced/Metastatic Solid Tumors or Lymphomas	IT	NCT03010176	2017	Completed
	II	Metastatic or Unresectable, Recurrent Head and Neck Squamous Cell Carcinoma	IT	NCT04220866	2020	Completed
GSK-3745417	I	Advanced Solid Tumors	IV	NCT03843359	2019	Active, not recruiting
BMS-986301	I	Advanced Solid Cancers	IV/IT/IM	NCT03956680	2019	Completed
SB-11285	I	Advanced Solid Tumors	IV	NCT04096638	2019	Completed
IMSA-101	I/II	Refractory Malignancies	IT	NCT04020185	2019	Completed
BI-1387446	I	Advanced or Metastatic Cancer	IT	NCT04147234	2020	Completed
TAK-676	I/II	Advanced or Metastatic Solid Tumors	IV	NCT04420884	2020	Completed
	I	A Number of Cancers	IV	NCT04879849	2021	Completed
eXOSTING	I/II	Advanced/​Metastatic, Recurrent, Injectable Solid Tumors	IT	NCT04592484	2020	Completed
HG-381	I	Advanced Solid Tumors	IV	NCT04998422	2021	Recruiting

Taken together, the clinical experience with IDO inhibitors and STING/TLR7/8 agonists identifies several translational challenges that are directly relevant to PDNP development, including limited therapeutic windows, insufficient or unsustained local immune modulation, and substantial variability in treatment response. These findings provide a clinical rationale, rather than direct clinical validation, for tumor-activated prodrug strategies. From this perspective, PDNPs may offer several design opportunities: prodrug masking and tumor-restricted activation may reduce systemic exposure to potent immunomodulators; coordinated delivery with treatments that induce ICD or remodel the TME may improve the spatial and temporal coupling of antigen release and immune stimulation; and biomarker-informed patient selection may become particularly important when prodrug activation depends on heterogeneous tumor-associated signals. Nevertheless, whether these theoretical advantages can translate into meaningful clinical benefit remains to be demonstrated in dedicated translational and clinical studies of PDNP formulations.

### 5.2 Translational barriers and design considerations for PDNPs

Building on the clinical lessons discussed above, the translational performance of PDNPs depends not only on the pharmacological activity of their therapeutic components, but also on the behavior of the nanoformulation throughout the delivery process. Despite differences in therapeutic payloads and activation mechanisms, PDNPs share several common requirements, including sufficient stability during systemic circulation, favorable biodistribution, effective tumor penetration, predictable tumor-localized activation, and limited exposure of normal tissues. Accordingly, this subsection focuses on the pharmacokinetic and biological barriers that should be considered when designing PDNPs for clinical translation. Pharmacokinetic behavior is a major determinant of the biodistribution, clearance, and toxicity profiles of PDNPs. Similar translational concerns have also been emphasized in recent analyses of nanomedicine-based metallodrugs, where nonspecific distribution, unfavorable pharmacokinetics, limited bioavailability, and potential toxicity remain major barriers to clinical application (119). Recent GSH-, ROS-, and hypoxia-responsive prodrug nanosystems further illustrate how stimulus-responsive nanoformulation can improve tumor selectivity and mitigate pharmacokinetic or off-target limitations of therapeutic agents (120–122). For instance, Shim et al. developed a doxorubicin prodrug nanoparticle (CatB-NPs) that is cleavable by cathepsin B. Compared with free doxorubicin, CatB-NPs prolonged the drug half-life 1.27-fold, increased the maximum blood concentration 53.73-fold, and increased the area under the concentration–time curve (AUC) 30.26-fold; the formulation also remained stable in plasma for 48 h (35). Therefore, precisely regulating pharmacokinetics is a crucial step for the clinical application of PDNPs. On the one hand, by selecting appropriate carrier materials, the circulation time of the drug can be extended, thereby enhancing the passive targeting efficiency of the tumor. For instance, Li et al. investigated the effect of varying the mass ratio of a docetaxel dimeric prodrug to DSPE-mPEG2000 on nanoassembly performance. At a prodrug-to-PEG mass ratio of 8:2, the resulting nanoparticles exhibited favorable structural stability. The AUC of total docetaxel was approximately 19.66-fold higher than that of the docetaxel solution, demonstrating the substantial influence of PEG content on systemic pharmacokinetics (123). On the other hand, it is necessary to avoid excessive prolongation of blood circulation, which could lead to the accumulation of drugs in non-target organs (such as the liver and spleen), thereby causing off-target toxicity. For instance, a study systematically investigated the impact of different levels of PEGylation on the *in vivo* behavior of SN38 PDNPs. The study showed that excessive PEGylation could compromise nanoparticle stability, with the 200% PEGylated formulation exhibiting particularly pronounced instability. By contrast, the 150% PEGylated formulation achieved a circulation half-life of 18.43 h without obvious toxicity in major off-target organs such as the liver and spleen, while reducing premature drug release and intestinal toxicity (124). These findings indicate that PEGylation should be optimized rather than simply maximized, because improvements in one pharmacokinetic parameter may be accompanied by trade-offs in other formulation properties.

Beyond systemic pharmacokinetics, nano–bio interfacial interactions also play a critical role in determining the delivery efficiency of PDNPs. After entering the bloodstream, nanoparticles encounter a series of biological barriers that influence their subsequent biodistribution and tumor delivery (125). To reach tumor tissues, nanoparticles must extravasate across the tumor vasculature while avoiding rapid clearance by phagocytic cells (126). Surface PEGylation is widely used to reduce nonspecific protein adsorption and phagocytic uptake (127). At the same time, formation of a protein corona can reshape the “biological identity” of nanoparticles and substantially influence their *in vivo* fate (128–131). Following extravasation, nanoparticles must further diffuse through the dense tumor extracellular matrix and ultimately be internalized by target cells to exert their therapeutic effects. However, reliance on passive accumulation and diffusion alone can limit both vascular extravasation and deep intratumoral penetration. Moreover, incorporating multiple stimulus-responsive features may improve activation specificity but simultaneously increases formulation complexity and may reduce the predictability of activation in a spatially and temporally heterogeneous TME (132). Against this background, Li et al. developed a series of amphiphilic polysulfobetaine–polycaprolactone micelles with tunable N-substituted alkyl-chain lengths. They found that the PSB4-PCL formulation with an intermediate butyl side chain maintained protein resistance while retaining sufficient affinity for cell membranes. This balance promoted rapid transcytosis-mediated extravasation and deep tumor penetration without requiring increasingly complex stimulus-responsive mechanisms (133).

Despite substantial advances in preclinical development, PDNPs for cancer immunotherapy continue to face several common translational bottlenecks, including systemic toxicity, insufficient selectivity, and unpredictable *in vivo* behavior. Although pharmacokinetic regulation and interface engineering can improve drug distribution and circulation behavior, these strategies cannot completely eliminate off-target activation or accumulation in non-target tissues. More importantly, the evidence supporting PDNPs in cancer immunotherapy remains predominantly preclinical, and their therapeutic advantages have yet to be validated directly in clinical settings. In addition, the spatial and temporal heterogeneity of the TME can make stimulus-dependent activation variable across tumor types, patients, and even different regions within the same lesion, thereby reducing the predictability of drug release and therapeutic efficacy. These challenges also illustrate that optimizing a single property is unlikely to be sufficient for clinical translation. Prolonged circulation, enhanced surface shielding, deeper tumor penetration, and increased activation specificity can each provide benefits, but excessive optimization of one feature may compromise another or increase formulation complexity. Therefore, clinically oriented PDNP design should emphasize a balanced combination of circulation stability, tumor accessibility, predictable activation, limited off-target exposure, and structural simplicity rather than maximizing any individual performance parameter.

### 5.3 AI/ML-enabled future perspectives for PDNP development

Artificial intelligence (AI) and machine learning (ML) provide new opportunities to navigate the multidimensional design space of nanomedicines, in which molecular structure, intermolecular interactions, formulation composition, and processing conditions collectively determine nanoparticle formation and therapeutic performance (134). This capability is particularly relevant to PDNPs because their development requires simultaneous optimization of prodrug chemistry, self-assembly behavior, stimulus responsiveness, drug loading, and formulation stability. Recent studies have demonstrated that ML can directly facilitate the prediction of small-molecule self-assembly. Zhang et al. constructed ML models using physicochemical descriptors of drug combinations and achieved an accuracy of 91.89% with the optimal logistic regression model for predicting the formation of small-molecule-assembled nanomedicines (135). AI-assisted virtual screening has also enabled exploration of much larger chemical spaces. Wang et al. evaluated nearly 20 million virtual ionizable lipids by predicting apparent pKa and mRNA delivery efficiency, followed by experimental validation of selected candidates (136). Similarly, the AGILE platform combined deep learning with high-throughput combinatorial chemistry, using experimental data from 1,200 ionizable lipids to guide screening within larger virtual libraries and identify cell-specific delivery candidates (137). These approaches suggest that AI/ML could help prioritize prodrug structures, responsive linkers, and co-assembly partners before extensive experimental synthesis and screening, thereby reducing the experimental search space during early-stage PDNP development.

Beyond molecular screening, AI/ML can be extended to the optimization of multicomponent formulations and their *in vivo* behavior. The COMET model was trained on the LANCE dataset, which contains more than 3,000 LNP formulations, to encode molecular structures, component ratios, and synthesis parameters, and was subsequently applied to screen 50 million virtual formulations. Notably, the model could also be adapted to different cell types and used to predict formulation stability after lyophilization, illustrating the potential of AI to optimize multiple formulation endpoints rather than efficacy alone (138). Computational frameworks such as EVONANO further integrate tumor growth, nanoparticle transport, and evolutionary optimization to identify favorable combinations of diffusion, binding affinity, concentration, and drug loading (139). In parallel, ML and AI-assisted physiologically based pharmacokinetic modeling have been explored for predicting nanoparticle pharmacokinetics, biodistribution, and safety (140–143), while large-scale analyses of preclinical nanomedicine datasets have revealed complex relationships among particle size, morphology, surface modification, targeting strategy, and therapeutic efficacy (144). For PDNPs, future models could further incorporate activation-specific descriptors, such as pH sensitivity, redox conditions, enzyme abundance, hypoxia, and drug-release kinetics, thereby jointly optimizing circulation stability, tumor accessibility, activation selectivity, and therapeutic efficacy.

AI/ML may also facilitate the development of more personalized PDNP formulations. The AGILE study revealed distinct ionizable-lipid preferences among different cell types, indicating that a universally optimized nanoparticle formulation may not necessarily provide optimal performance across different biological contexts (137). More broadly, AI-driven drug-delivery frameworks have proposed integrating molecular profiling, targeting information, physicochemical properties, pharmacokinetic modeling, and patient-specific biological data throughout the nanocarrier design process (145). Applied to PDNPs, such approaches could potentially integrate tumor-specific information on acidity, enzyme expression, redox status, hypoxia, molecular targets, and imaging characteristics to support the selection of appropriate activation mechanisms, targeting strategies, and combination regimens. However, the direct application of AI/ML to PDNP development remains at an early stage because most currently available datasets originate from LNPs or other classes of nanomedicines rather than tumor-activated prodrug nanoparticles. Model performance is therefore strongly dependent on the size, diversity, and quality of training data, and limited generalizability remains an important challenge (136, 137, 145). In addition, model interpretability, standardized data reporting, and experimental validation remain essential for reliable translation. Future progress will require standardized datasets that integrate prodrug structures, linker chemistry, formulation parameters, activation kinetics, pharmacokinetics, and therapeutic outcomes. Accordingly, the near-term role of AI/ML in PDNP development is more likely to involve candidate prioritization, reduction of experimental search space, pharmacokinetic optimization, and patient stratification rather than replacement of mechanistic and experimental studies.

### 5.4 Future directions toward clinical translation

Despite substantial progress in preclinical studies, several challenges must be addressed before PDNPs can achieve broader clinical translation. First, future PDNP design should move beyond maximizing stimulus responsiveness and instead balance biosafety, circulation stability, tumor accessibility, activation selectivity, and formulation reproducibility. Nanoparticle performance is strongly influenced by biological barriers encountered during tissue trafficking, cellular uptake, and subcellular delivery, making rational control of these processes essential for improving tumor targeting while minimizing off-target toxicity (146). Pharmacokinetic behavior should also be considered at the earliest stages of formulation development, because nanoparticle composition, size, surface properties, structural stability, and drug-release kinetics collectively influence blood circulation, biodistribution, and clearance. In addition, differences between animal models and humans, nonspecific accumulation in clearance organs such as the liver and spleen, and batch-to-batch variability remain important barriers to clinical translation (147). Accordingly, formulation design should be integrated with optimization of dose, administration route, treatment sequence, pharmacokinetics, and biodistribution rather than evaluated as an isolated material-development problem.

The substantial inter-patient and intratumoral heterogeneity of the TME makes it unlikely that a single activation threshold or targeting strategy will be optimal for all tumors. Future PDNP development should therefore incorporate patient-specific tumor characteristics when selecting responsive mechanisms and targeting strategies. In this context, advanced patient-derived tumor models may provide a useful bridge between conventional preclinical testing and individualized treatment. Compared with conventional epithelial-only organoids, more complex organoid systems incorporating immune and stromal components, including co-culture models, air–liquid interface cultures, and patient-derived organotypic tumor spheroids, can better reproduce important features of the patient-specific TME and its response to therapy (148). Patient-derived organoids have also been increasingly explored for molecular profiling, drug screening, biomarker discovery, and prediction of treatment response, supporting their potential use in precision cancer medicine (149). For PDNPs, such platforms could eventually be used to compare candidate formulations according to activation behavior, therapeutic efficacy, and immune effects in patient-specific tumor environments. However, this application remains prospective, and model representativeness, reproducibility, throughput, and correlation with clinical outcomes will need to be established before organoid-guided PDNP selection can be incorporated into translational workflows. Overall, successful clinical translation will likely depend on integrating rational formulation design, pharmacokinetic optimization, clinically relevant disease models, and patient stratification rather than simply increasing formulation complexity.

## 6 Conclusions

Tumor-activated PDNPs represent a versatile strategy for improving the spatial and temporal control of cancer immunotherapy by integrating prodrug chemistry with nanoscale delivery. Current designs exploit endogenous tumor-associated cues, including acidity, redox conditions, enzymatic activity, and hypoxia, as well as externally applied stimuli such as light and ultrasound, to restrict therapeutic activation to defined biological or physical environments. Active targeting can further enhance tumor- or cell-specific localization, while hybrid and multistage systems combine targeting and stimulus-responsive mechanisms to achieve more stringent control over drug delivery and activation. Beyond reducing premature systemic exposure, these platforms can coordinate chemotherapy, photodynamic or sonodynamic therapy, and immune modulation to induce immunogenic cell death, promote dendritic cell activation and T-cell infiltration, and enhance responses to immune checkpoint blockade. Nevertheless, increasing functional complexity does not necessarily translate into superior therapeutic performance. The overall value of a PDNP depends on achieving an appropriate balance among circulation stability, tumor accessibility, activation selectivity, therapeutic efficacy, and formulation reproducibility. Thus, the continued development of PDNPs should emphasize not only responsive activation itself, but also the robustness and translational relevance of the complete delivery–activation process.
